# Supplementary material for: Comparative genomics of Lentilactobacillus buchneri reveals strain-level hyperdiversity and broad-spectrum CRISPR immunity against human and livestock gut phages
Source: PLoS One. 2025 Jun 10;20(6):e0325832. doi: 10.1371/journal.pone.0325832 (PMC12151389; doi:10.1371/journal.pone.0325832)
Supplement: S1 Table — (PDF) [file pone.0325832.s001.pdf]

S1 Table. CRISPRCasFinder results of *L. buchneri* genomes tested

Strain	Element	CRISPR Id / Cas Type	Start	End	Spacer / Gene	Repeat consensus / cas genes	Direction	Evidence Level
177	CRISPR	177_1	828858	828960	1	CCCCCTTATATGGCCGGAAGCTGC	ND	1
177	Cas cluster	CAS-TypeIIA	862555	868734	4	cas9_TypeII, cas1_TypeII, cas2_TypeI-II-III, csn2_TypeIIA		
177	CRISPR	177_2	868763	869855	16	GTTTTAGAAGGATGTTAAATCAATAAGGTTAAACCC	ND	4
177	CRISPR	177_3	1831775	1832415	10	GTATTCCCCACGTACGTAGGGGTGATCC	-	4
177	Cas cluster	CAS-TypeIE	1832786	1842238	8	cas3_TypeI, cse1_TypeIE, cse2_TypeIE, cas7_TypeIE, cas5_TypeIE, cas6_TypeIE, cas1_TypeIE, cas2_TypeIE		
177	CRISPR	177_4	1842268	1842967	11	GTATTCCCCACGTACGTAGGGGTGATCC	-	4
177	CRISPR	177_5	1844188	1845744	25	GTATTCCCCACGTACGTAGGGGTGATCC	-	4
177	CRISPR	177_6	1848708	1848918	3	GTATTCCCCACGTGTGTAGGGGTGATCC	-	1
177	CRISPR	177_7	2136632	2136774	1	GGAAACCGAGTCAGGTTTTCGGCTGGCGGCGCTTAAATTTCAATTGCTGTCCT	ND	1
1012	CRISPR	1012_1	1493200	1494093	13	GTTTTAGAAGGATGTTAAATCAATAAGGTTAAACCC	ND	4
1012	Cas cluster	CAS-TypeIIA	1658070	1664248	4	cas9_TypeII, cas1_TypeII, cas2_TypeI-II-III, csn2_TypeIIA		
1012	CRISPR	1012_2	1664277	1664708	6	GTTTTAGAAGGATGTTAAATCAATAAGGTTAAACCC	ND	4
1012	CRISPR	1012_3	1719929	1720085	1	TGAACCCCTTGGTACAAGCGGACTTCTCAAAAAGAGA	ND	1
1014	CRISPR	1014_1	1162406	1163894	22	GGGTTTAACCTTATTGATTTAACATCCTTCTAAAAC	-	4
1014	Cas cluster	CAS-TypeIIA	1163923	1170101	4	csn2_TypeIIA, cas2_TypeI-II-III, cas1_TypeII, cas9_TypeII		
1014	CRISPR	1014_2	1187622	1187750	1	CTTGCTGTCCGCTTTCACACGTTTGATCCAAATAACAGT	-	1
ATCC 4005	CRISPR	ATCC_4005_1	696227	696856	9	GGGTTTAACCTTATTGATTTAACATCCTTCTAAAAC	-	4

ATCC 4005	Cas cluster	CAS-TypeIIA	696885	703063	4	csn2_TypeIIA, cas2_TypeI-II-III, cas1_TypeII, cas9_TypeII		
ATCC 4005	CRISPR	ATCC_4005_2	720708	720836	1	CTTGCTGTCCGCTTTCACACGTTTGATCCAAATAACAGT	-	1
ATCC 4005	CRISPR	ATCC_4005_3	2523840	2523989	1	GTAAATAAAATTTACTTGTAAATAAAGTTTACTTGTAAATATTATTTAC	ND	1
CD034	CRISPR	CD034_1	115858	116559	11	GTATTCCCCACGTACGTAGGGGTGATCC	-	4
CD034	Cas cluster	CAS-TypeIE	116930	126382	8	cas3_TypeI, cse1_TypeIE, cse2_TypeIE, cas7_TypeIE, cas5_TypeIE, cas6_TypeIE, cas1_TypeIE, cas2_TypeIE		
CD034	CRISPR	CD034_2	126412	127111	11	GTATTCCCCACGTACGTAGGGGTGATCC	-	4
CD034	CRISPR	CD034_3	128332	129888	25	GTATTCCCCACGTACGTAGGGGTGATCC	-	4
CD034	CRISPR	CD034_4	132852	133062	3	GTATTCCCCACGTGTGTAGGGGTGATCC	-	1
CD034	CRISPR	CD034_5	420773	420915	1	GGAAACCGAGTCAGGTTTTCGGCTGGCGGCGCTTAAATTTCAATTGCTGTCCT	ND	1
CD034	CRISPR	CD034_6	1941773	1942865	16	GGGTTTAACCTTATTGATTTAACATCCTTCTAAAAC	-	4
CD034	Cas cluster	CAS-TypeIIA	1942894	1949073	4	csn2_TypeIIA, cas2_TypeI-II-III, cas1_TypeII, cas9_TypeII		
CD034	CRISPR	CD034_7	1982668	1982770	1	GCAGCTTCCGGCCATATAAGGGGG	ND	1
CIRM-BIA 1514	CRISPR	CIRM-BIA_1514_1	1375564	1375706	1	AGGACAGCAATTGAAATTTAAGCGCCGCCAGCCGAAAACCTGACTCGGTTTCC	ND	1
CIRM-BIA 1514	Cas cluster	CAS-TypeIE	1746473	1753400	5	cas5_TypeIE, cas7_TypeIE, cse2_TypeIE, cse1_TypeIE, cas3_TypeI		
CIRM-BIA 1514	CRISPR	CIRM-BIA_1514_2	1753770	1754348	9	AGGATCACCCCTACGTACGTGGGGAATAC	ND	4
CIRM-BIA 1514	Cas cluster	CAS-TypeIE	2065992	2068213	3	cas6_TypeIE, cas1_TypeIE, cas2_TypeIE		
CIRM-BIA 1514	CRISPR	CIRM-BIA_1514_3	2068243	2068943	11	GTATTCCCCACGTACGTAGGGGTGATCCT	ND	4
CIRM-BIA 1514	CRISPR	CIRM-BIA_1514_4	2070163	2071659	24	GTATTCCCCACGTACGTAGGGGTGATCCT	ND	4

CIRM-BIA 1514	CRISPR	CIRM-BIA_1514_5	2074622	2074832	3	GTATTCCCCACGTGTGTAGGGGTGATCC	-	1
CIRM-BIA 1514	CRISPR	CIRM-BIA_1514_6	2473987	2474089	1	CCCCCTTATATGGCCGGAAGCTGC	ND	1
CIRM-BIA 1514	Cas cluster	CAS-TypeIIA	2507684	2513862	4	cas9_TypeII, cas1_TypeII, cas2_TypeI-II-III, csn2_TypeIIA		
CIRM-BIA 1514	CRISPR	CIRM-BIA_1514_7	2513891	2514983	16	GTTTTAGAAGGATGTTAAATCAATAAGGTTAAACCC	ND	4
CIRM-BIA 1516	CRISPR	CIRM-BIA_1516_1	141346	142438	16	GGGTTTAACCTTATTGATTTAACATCCTTCTAAAAC	-	4
CIRM-BIA 1516	Cas cluster	CAS-TypeIIA	142467	148646	4	csn2_TypeIIA, cas2_TypeI-II-III, cas1_TypeII, cas9_TypeII		
CIRM-BIA 1516	CRISPR	CIRM-BIA_1516_2	182241	182343	1	GCAGCTTCCGGCCATATAAGGGGG	ND	1
CIRM-BIA 1516	CRISPR	CIRM-BIA_1516_3	965937	966079	1	AGGACAGCAATTGAAATTTAAGCGCCGCCAGCCGAAAACCTGACTCGGTTTCC	ND	1
CIRM-BIA 1516	CRISPR	CIRM-BIA_1516_4	1803996	1804206	3	GGATCACCCCTACACACGTGGGGAATAC	ND	1
CIRM-BIA 1516	CRISPR	CIRM-BIA_1516_5	1807170	1808667	24	GGATCACCCCTACGTACGTGGGGAATAC	ND	4
CIRM-BIA 1516	CRISPR	CIRM-BIA_1516_6	1809888	1810586	11	GGATCACCCCTACGTACGTGGGGAATAC	ND	4
CIRM-BIA 1516	Cas cluster	CAS-TypeIE	1810616	1820068	8	cas2_TypeIE, cas1_TypeIE, cas6_TypeIE, cas5_TypeIE, cas7_TypeIE, cse2_TypeIE, cse1_TypeIE, cas3_TypeI		
CIRM-BIA 1516	CRISPR	CIRM-BIA_1516_7	1820439	1821140	11	GGATCACCCCTACGTACGTGGGGAATAC	ND	4
CIRM-BIA 2081	CRISPR	CIRM-BIA_2081_1	326667	327825	17	GGGTTTAACCTTATTGATTTAACATCCTTCTAAAAC	-	4
CIRM-BIA 2081	Cas cluster	CAS-TypeIIA	327854	334033	4	csn2_TypeIIA, cas2_TypeI-II-III, cas1_TypeII, cas9_TypeII		

CIRM-BIA 2081	CRISPR	CIRM-BIA_2081_2	367628	367730	1	GCAGCTTCCGGCCATATAAGGGGG	ND	1
CIRM-BIA 2081	CRISPR	CIRM-BIA_2081_3	1432041	1432183	1	GGAAACCGAGTCAGGTTTTCGGCTGGCGGCGCTTAAATTTCAATTGCTGTCCT	ND	1
CIRM-BIA 2081	CRISPR	CIRM-BIA_2081_4	2581180	2581390	3	GGATCACCCCTACACACGTGGGGAATAC	ND	1
CIRM-BIA 2081	CRISPR	CIRM-BIA_2081_5	2584354	2585911	25	GGATCACCCCTACGTACGTGGGGAATAC	ND	4
CIRM-BIA 2081	CRISPR	CIRM-BIA_2081_6	2587132	2587830	11	GGATCACCCCTACGTACGTGGGGAATAC	ND	4
CIRM-BIA 2081	Cas cluster	CAS-TypeIE	2587860	2597312	8	cas2_TypeIE, cas1_TypeIE, cas6_TypeIE, cas5_TypeIE, cas7_TypeIE, cse2_TypeIE, cse1_TypeIE, cas3_TypeI		
CIRM-BIA 2081	CRISPR	CIRM-BIA_2081_7	2597683	2598384	11	GGATCACCCCTACGTACGTGGGGAATAC	ND	4
CIRM-BIA 2082	CRISPR	CIRM-BIA_2082_1	2337790	2339409	24	GGGTTTAACCTTATTGATTTAACATCCTTCTAAAAC	-	4
CIRM-BIA 2082	Cas cluster	CAS-TypeIIA	2339438	2345616	4	csn2_TypeIIA, cas2_TypeI-II-III, cas1_TypeII, cas9_TypeII		
CIRM-BIA 2083	CRISPR	CIRM-BIA_2083_1	1364170	1364271	1	GTCTATTTTTAGACTTTCGCTCGTAGCAAGGGTTT	ND	1
CIRM-BIA 2083	CRISPR	CIRM-BIA_2083_2	1378674	1380425	26	GGGTTTAACCTTATTGATTTAACATCCTTCTAAAAC	-	4
CIRM-BIA 2083	Cas cluster	CAS-TypeIIA	1380454	1386632	4	csn2_TypeIIA, cas2_TypeI-II-III, cas1_TypeII, cas9_TypeII		
CIRM-BIA 2083	CRISPR	CIRM-BIA_2083_3	2213194	2213330	1	AAAGCTAAGCCATTCCTGCCGAAAACCTGTCTCGGTTTCCGTCC	ND	1
CIRM-BIA 2084	CRISPR	CIRM-BIA_2084_1	198682	198831	1	GTAAATAAAATTTACTTGTAAATAAAGTTTACTTGTAAATATTATTTAC	ND	1
CIRM-BIA 2084	CRISPR	CIRM-BIA_2084_2	417319	417455	1	GGACGGAAACCGAGTCAGGTTTTCGGCAGGAATGGCTTAGCTTT	ND	1

CIRM-BIA 2084	Cas cluster	CAS-TypeIIA	1744828	1751006	4	cas9_TypeII, cas1_TypeII, cas2_TypeI-II-III, csn2_TypeIIA		
CIRM-BIA 2084	CRISPR	CIRM-BIA_2084_3	1751035	1752786	26	GTTTTAGAAGGATGTTAAATCAATAAGGTTAAACCC	ND	4
CIRM-BIA 659	CRISPR	CIRM-BIA_659_1	1197607	1198236	9	GGGTTTAACCTTATTGATTTAACATCCTTCTAAAAC	-	4
CIRM-BIA 659	Cas cluster	CAS-TypeIIA	1198265	1204443	4	csn2_TypeIIA, cas2_TypeI-II-III, cas1_TypeII, cas9_TypeII		
CIRM-BIA 659	CRISPR	CIRM-BIA_659_2	1222088	1222216	1	CTTGCTGTCCGCTTTCACACGTTTGATCCAAATAACAGT	-	1
CIRM-BIA 664	CRISPR	CIRM-BIA_664_1	205327	205434	1	AATTGTCAAATCAAGTGCAACACTA	ND	1
CIRM-BIA 664	CRISPR	CIRM-BIA_664_2	1829901	1830530	9	GGGTTTAACCTTATTGATTTAACATCCTTCTAAAAC	-	4
CIRM-BIA 664	Cas cluster	CAS-TypeIIA	1830559	1836737	4	csn2_TypeIIA, cas2_TypeI-II-III, cas1_TypeII, cas9_TypeII		
CIRM-BIA 664	CRISPR	CIRM-BIA_664_3	1854382	1854510	1	CTTGCTGTCCGCTTTCACACGTTTGATCCAAATAACAGT	-	1
CIRM-BIA 845	CRISPR	CIRM-BIA_845_1	1190748	1191377	9	GGGTTTAACCTTATTGATTTAACATCCTTCTAAAAC	-	4
CIRM-BIA 845	Cas cluster	CAS-TypeIIA	1191406	1197584	4	csn2_TypeIIA, cas2_TypeI-II-III, cas1_TypeII, cas9_TypeII		
CIRM-BIA 845	CRISPR	CIRM-BIA_845_2	1215229	1215357	1	CTTGCTGTCCGCTTTCACACGTTTGATCCAAATAACAGT	-	1
DSM 20057	CRISPR	DSM_20057_1	1596918	1597547	9	GGGTTTAACCTTATTGATTTAACATCCTTCTAAAAC	-	4
DSM 20057	Cas cluster	CAS-TypeIIA	1597576	1603754	4	csn2_TypeIIA, cas2_TypeI-II-III, cas1_TypeII, cas9_TypeII		
DSM 20057	CRISPR	DSM_20057_2	1921301	1921450	1	GTAAATAAAATTTACTTGTAAATAAAGTTTACTTGTAAATATTATTTAC	ND	1
FUA3252	CRISPR	FUA3252_1	6265	6369	1	TGTCCGATTTTCGGATTTTCGCTCGTAGCAAGGGTTTG	ND	1

FUA3252	CRISPR	FUA3252_2	12763	12864	1	GTCTATTTTTAGACTTTCGCTCGTAGCAAGGGTTT	ND	1
FUA3252	CRISPR	FUA3252_3	92193	92296	1	AACTGCATTTTTGCAGTTCCGCTTGTACCAAGGGATT	ND	1
FUA3252	Cas cluster	CAS-TypeIIA	534202	540380	4	cas9_TypeII, cas1_TypeII, cas2_TypeI-II-III, csn2_TypeIIA		
FUA3252	CRISPR	FUA3252_4	540409	541566	17	GTTTTAGAAGGATGTTAAATCAATAAGGTTAAACCC	ND	4
FUA3252	CRISPR	FUA3252_5	1792123	1792255	1	ATTGGATTTTAAGCCCCTCCACCCGAAAACCTGACTCGGTTTC	ND	1
LA1147	CRISPR	LA1147_1	74319	74422	1	AACTGCATTTTTGCAGTTCCGCTTGTACCAAGGGATT	ND	1
LA1147	CRISPR	LA1147_2	185938	186095	1	AAACCCTTGCTACGAGCGGAAGTTCAAAAAATGAACTT	ND	1
LA1147	CRISPR	LA1147_3	1553084	1553190	1	GGATAGTTTCTATCCTTTGGAGATCTCCTCGTGTGGG	ND	1
LA1147	CRISPR	LA1147_4	2026526	2027617	16	GGGTTTAACCTTATTGATTTAACATCCTTCTAAAAC	-	4
LA1147	Cas cluster	CAS-TypeIIA	2027646	2033824	4	csn2_TypeIIA, cas2_TypeI-II-III, cas1_TypeII, cas9_TypeII		
LA1147	CRISPR	LA1147_5	2152926	2153081	1	AAACCCTTGCTGCGCGCAGAACTGTAAATTCTACAG	ND	1
LA1161B	CRISPR	LA1161B_1	16672	16827	1	AAACCCTTGCTACGAGCGAAAGTCTAAAAAATAGAC	ND	1
LA1161B	CRISPR	LA1161B_2	188823	188980	1	CAAACCCTTGCTACGAGCGAAAATCCGAAAATCGGACA	ND	1
LA1161B	CRISPR	LA1161B_3	872981	873113	1	GAAACCGAGTCAGGTTTTCGGCTGGAGGGGCTTAAAATCCAAT	ND	1
LA1161B	CRISPR	LA1161B_4	2069764	2071779	30	GGGTTTAACCTTATTGATTTAACATCCTTCTAAAAC	-	4
LA1161B	Cas cluster	CAS-TypeIIA	2071808	2077986	4	csn2_TypeIIA, cas2_TypeI-II-III, cas1_TypeII, cas9_TypeII		
LA1161C	CRISPR	LA1161C_1	37184	37285	1	GTCTATTTTTAGACTTTCGCTCGTAGCAAGGGTTT	ND	1
LA1161C	CRISPR	LA1161C_2	130350	130507	1	CAAACCCTTGCTACGAGCGAAAATCCGAAAATCGGACA	ND	1
LA1161C	CRISPR	LA1161C_3	160193	160324	1	GACTGAAAATATTAAGCCTAACTTCGCCGAGATTGCCCGGC	ND	1
LA1161C	CRISPR	LA1161C_4	831411	831543	1	GAAACCGAGTCAGGTTTTCGGCTGGAGGGGCTTAAAATCCAAT	ND	1

LA1161C	CRISPR	LA1161C_5	2019337	2021352	30	GGGTTTAACCTTATTGATTTAACATCCTTCTAAAAC	-	4
LA1161C	Cas cluster	CAS-TypeIIA	2021381	2027559	4	csn2_TypeIIA, cas2_TypeI-II-III, cas1_TypeII, cas9_TypeII		
LA1167	CRISPR	LA1167_1	97165	97322	1	CAAACCCTTGCTACGAGCGAAAATCCGAAAATCGGACA	ND	1
LA1167	CRISPR	LA1167_2	161162	161725	8	GGGTTTAACCTTATTGATTTAACATCCTTCTAAAAC	-	4
LA1167	CRISPR	LA1167_3	182852	182953	1	GTCTATTTTTAGACTTTCGCTCGTAGCAAGGGTTT	ND	1
LA1167	CRISPR	LA1167_4	860757	860889	1	GAAACCGAGTCAGGTTTTCGGCTGGAGGGGCTTAAAATCCAAT	ND	1
LA1167	CRISPR	LA1167_5	2049967	2051982	30	GGGTTTAACCTTATTGATTTAACATCCTTCTAAAAC	-	4
LA1167	Cas cluster	CAS-TypeIIA	2052011	2058189	4	csn2_TypeIIA, cas2_TypeI-II-III, cas1_TypeII, cas9_TypeII		
LA1167	CRISPR	LA1167_6	2070557	2070724	2	GGGTTTAACCTTATTGATTTAACATCCTTCTAAAAC	-	1
LA1167	Cas cluster	CAS-TypeIIA	2070753	2076924	4	csn2_TypeIIA, cas2_TypeI-II-III, cas1_TypeII, cas9_TypeII		
LA1175D	CRISPR	LA1175D_1	137132	137233	1	GTCTATTTTTAGACTTTCGCTCGTAGCAAGGGTTT	ND	1
LA1175D	CRISPR	LA1175D_2	158596	158697	1	CTGTAGAATTTACAGTTCTGCGCGCAGCAAGGGTTT	ND	1
LA1175D	CRISPR	LA1175D_3	1225732	1225828	1	TCAACACCATTTGACAAACTTCCCAAA	ND	1
LA1175D	CRISPR	LA1175D_4	2124570	2125661	16	GGGTTTAACCTTATTGATTTAACATCCTTCTAAAAC	-	4
LA1175D	Cas cluster	CAS-TypeIIA	2125690	2131868	4	csn2_TypeIIA, cas2_TypeI-II-III, cas1_TypeII, cas9_TypeII		
LA1181	CRISPR	LA1181_1	132375	132533	1	TCAATCCCTTGCTACGAGCGGAATTTCGAAAATCGAAAG	ND	1
LA1181	CRISPR	LA1181_2	162594	162695	1	GTCCGATTTTCGGATTTTCGCTCGTAGCAAGGGTTT	ND	1
LA1181	CRISPR	LA1181_3	198924	199079	1	AAACCCTTGCTACGAGCGAAAGTCTAAAAAATAGAC	ND	1
LA1181	CRISPR	LA1181_4	382202	382320	1	CTGCTTATTTGCCCACGATTCCACTAGGTAATAGT	ND	1
LA1181	CRISPR	LA1181_5	2110677	2112362	25	GGGTTTAACCTTATTGATTTAACATCCTTCTAAAAC	-	4

LA1181	Cas cluster	CAS-TypeIIA	2112391	2118569	4	csn2_TypeIIA, cas2_TypeI-II-III, cas1_TypeII, cas9_TypeII		
LA1184	CRISPR	LA1184_1	1833	1988	1	AAACCCTTGCTACGAGCGAAAATCCGAAAATCGGAC	ND	1
LA1184	CRISPR	LA1184_2	153890	153973	1	GGTATTCCCCACGTATGTGGGGG	ND	1
LA1184	CRISPR	LA1184_3	1606207	1606325	1	ACTATTACCTAGTGGAATCGTGGGCAAATCAGCAG	ND	1
LA1184	CRISPR	LA1184_4	2364464	2364593	1	ACTGTTATTTGGATCAAACGTGTGAAAGCGGACAGCAAGC	ND	1
LA1184	Cas cluster	CAS-TypeIIA	2383324	2389502	4	cas9_TypeII, cas1_TypeII, cas2_TypeI-II-III, csn2_TypeIIA		
LA1184	CRISPR	LA1184_5	2389531	2390820	19	GTTTTAGAAGGATGTTAAATCAATAAGGTTAAACCC	ND	4
MGB0786	CRISPR	MGB0786_1	347830	348789	14	GGGTTTAACCTTATTGATTTAACATCCTTCTAAAAC	-	4
MGB0786	Cas cluster	CAS-TypeIIA	348818	356033	4	csn2_TypeIIA, cas2_TypeI-II-III, cas1_TypeII, cas9_TypeII		
MGB0786	CRISPR	MGB0786_2	1589128	1589260	1	GAAACCGAGTCAGGTTTTCGGCTGGAGGGGCTTAAAATCCAAT	ND	1
MGR2-32	CRISPR	MGR2-32_1	350102	350600	7	GGGTTTAACCTTATTGATTTAACATCCTTCTAAAAC	-	4
MGR2-32	Cas cluster	CAS-TypeIIA	350629	356808	4	csn2_TypeIIA, cas2_TypeI-II-III, cas1_TypeII, cas9_TypeII		
MGR2-32	CRISPR	MGR2-32_2	390403	390505	1	GCAGCTTCCGGCCATATAAGGGGG	ND	1
MGR2-32	CRISPR	MGR2-32_3	1024083	1024784	11	GTATTCCCCACGTACGTAGGGGTGATCC	-	4
MGR2-32	Cas cluster	CAS-TypeIE	1025155	1034607	8	cas3_TypeI, cse1_TypeIE, cse2_TypeIE, cas7_TypeIE, cas5_TypeIE, cas6_TypeIE, cas1_TypeIE, cas2_TypeIE		
MGR2-32	CRISPR	MGR2-32_4	1034637	1035336	11	GTATTCCCCACGTACGTAGGGGTGATCC	-	4
MGR2-32	CRISPR	MGR2-32_5	1036557	1038050	24	GTATTCCCCACGTACGTAGGGGTGATCC	-	4
MGR2-32	CRISPR	MGR2-32_6	1041014	1041224	3	GTATTCCCCACGTGTGTAGGGGTGATCC	-	1
MGR2-32	CRISPR	MGR2-32_7	1368063	1368205	1	GGAAACCGAGTCAGGTTTTCGGCTGGCGGCGCTTAAATTTCAATTGCTGTCCT	ND	1

NBRC 107764	CRISPR	NBRC_107764_1	986285	986405	1	CCTCCAGCCAAAAGCCTGACTCGGCTTTCGTC	ND	1
NBRC 107764	CRISPR	NBRC_107764_2	1737267	1737395	1	ACTGTTATTTGGATCAAACGTGTGAAAGAGGACAGCAAG	ND	1
NBRC 107764	Cas cluster	CAS-TypeIIA	1755040	1761218	4	cas9_TypeII, cas1_TypeII, cas2_TypeI-II-III, csn2_TypeIIA		
NBRC 107764	CRISPR	NBRC_107764_3	1761247	1761876	9	GTTTTAGAAGGATGTTAAATCAATAAGGTTAAACCC	ND	4
NK01	CRISPR	NK01_1	241615	241757	1	AGGACAGCAATTGAAATTTAAGCGCCGCCAGCCGAAAACCTGACTCGGTTTCC	ND	1
NK01	CRISPR	NK01_2	776194	776343	2	TGTATTCCCCACGTACGTAGGGGTGATC	ND	1
NK01	Cas cluster	CAS-TypeIE	777267	786719	8	cas3_TypeI, cse1_TypeIE, cse2_TypeIE, cas7_TypeIE, cas5_TypeIE, cas6_TypeIE, cas1_TypeIE, cas2_TypeIE		
NK01	CRISPR	NK01_3	787176	787263	1	GTATTCCCCACGTACGTAGGGGTGAT	ND	1
NK01	CRISPR	NK01_4	788486	788573	1	GTATTCCCCACGTACGTAGGGGTGAT	ND	1
NK01	CRISPR	NK01_5	789658	789991	5	GTATTCCCCACGTACGTAGGGGTGAT	ND	2
NK01	CRISPR	NK01_6	792896	793106	3	GTATTCCCCACGTGTGTAGGGGTGATCC	-	1
NK01	CRISPR	NK01_7	1568655	1569022	5	GGGTTTAACCTTATTGATTTAACATCCTTCTAAAAC	-	4
NK01	CRISPR	NK01_8	1569316	1569483	2	GGGTTTAACCTTATTGATTTAACATCCTTCTAAAAC	-	1
NK01	Cas cluster	CAS-TypeIIA	1569512	1575691	4	csn2_TypeIIA, cas2_TypeI-II-III, cas1_TypeII, cas9_TypeII		
NK01	CRISPR	NK01_9	1609286	1609388	1	GCAGCTTCCGGCCATATAAGGGGG	ND	1
NRRL B-30929	CRISPR	NRRL_B-30929_1	671205	671334	1	GAAACCGAGTCAGGTTTTCGGCTGGAGGGGCTTAAAATCC	+	1
NRRL B-30929	CRISPR	NRRL_B-30929_2	1943645	1945330	25	GGGTTTAACCTTATTGATTTAACATCCTTCTAAAAC	-	4
NRRL B-30929	Cas cluster	CAS-TypeIIA	1945359	1951537	4	csn2_TypeIIA, cas2_TypeI-II-III, cas1_TypeII, cas9_TypeII		

NRRL B-30929	CRISPR	NRRL_B-30929_3	1969058	1969186	1	CTTGCTGTCCGCTTTCACACGTTTGATCCAAATAACAGT	-	1
NRRL B-30929	CRISPR	NRRL_B-30929_4	2578425	2578582	1	CAAACCCTTGCTACGAGCGAAAATCCGAAAATCGGACA	ND	1
PC-C1	CRISPR	PC-C1_1	1784765	1784885	1	CCTCCAGCCAAAAGCCTGACTCGGCTTTCGTC	ND	1
PC-C1	CRISPR	PC-C1_2	1914299	1915324	15	GGGTTTAACCTTATTGATTTAACATCCTTCTAAAAC	-	4
PC-C1	Cas cluster	CAS-TypeIIA	1915353	1921531	4	csn2_TypeIIA, cas2_TypeI-II-III, cas1_TypeII, cas9_TypeII		
RUG14303	CRISPR	RUG14303_1	1920925	1921053	1	ACTGTTATTTGGATCAAACGTGTGAAAGAGGACAGCAAG	ND	1
RUG14303	Cas cluster	CAS-TypeIIA	1949452	1955630	4	cas9_TypeII, cas1_TypeII, cas2_TypeI-II-III, csn2_TypeIIA		
RUG14303	CRISPR	RUG14303_2	1955659	1956024	5	GTTTTAGAAGGATGTTAAATCAATAAGGTTAAACCC	ND	3
S42	CRISPR	S42_1	168467	168591	1	TGTGAAAGAGGACAGCCATCGAAATTTAAGCCGCT	ND	1
S42	CRISPR	S42_2	228053	229146	16	GGGTTTAACCTTATTGATTTAACATCCTTCTAAAAC	-	4
S42	CRISPR	S42_3	236261	236417	1	TGAACCCCTTGGTACAAGCGGACTTCTCAAAAAGAGA	ND	1
S42	CRISPR	S42_4	631900	632601	11	GTATTCCCCACGTACGTAGGGGTGATCC	-	4
S42	Cas cluster	CAS-TypeIE	632972	642424	8	cas3_TypeI, cse1_TypeIE, cse2_TypeIE, cas7_TypeIE, cas5_TypeIE, cas6_TypeIE, cas1_TypeIE, cas2_TypeIE		
S42	CRISPR	S42_5	642454	643153	11	GTATTCCCCACGTACGTAGGGGTGATCC	-	4
S42	CRISPR	S42_6	644374	645442	17	GTATTCCCCACGTACGTAGGGGTGATCC	-	4
S42	CRISPR	S42_7	648406	648616	3	GTATTCCCCACGTGTGTAGGGGTGATCC	-	1
S42	CRISPR	S42_8	1441064	1441206	1	GGAAACCGAGTCAGGTTTTCGGCTGGCGGCGCTTAAATTTCAATTGCTGTCCT	ND	1
S42	CRISPR	S42_9	1682750	1682871	1	ACTCGGTTTCCTGGTGTCTTACTTTTATTTGG	ND	1
S42	CRISPR	S42_10	2441025	2441127	1	GCAGCTTCCGGCCATATAAGGGGG	ND	1
S43	CRISPR	S43_1	56562	56664	1	CCCCCTTATATGGCCGGAAGCTGC	ND	1

S43	CRISPR	S43_2	293173	294266	16	GTTTTAGAAGGATGTTAAATCAATAAGGTTAAACCC	ND	4
S43	CRISPR	S43_3	705755	706456	11	GTATTCCCCACGTACGTAGGGGTGATCC	-	4
S43	Cas cluster	CAS-TypeIE	706827	716279	8	cas3_TypeI, cse1_TypeIE, cse2_TypeIE, cas7_TypeIE, cas5_TypeIE, cas6_TypeIE, cas1_TypeIE, cas2_TypeIE		
S43	CRISPR	S43_4	716309	717008	11	GTATTCCCCACGTACGTAGGGGTGATCC	-	4
S43	CRISPR	S43_5	718229	719297	17	GTATTCCCCACGTACGTAGGGGTGATCC	-	4
S43	CRISPR	S43_6	722261	722471	3	GTATTCCCCACGTGTGTAGGGGTGATCC	-	1
S43	CRISPR	S43_7	1059297	1059446	1	GTAAATAAAATTTACTTGTAAATAAAGTTTACTTGTAAATATTATTTAC	ND	1
S43	CRISPR	S43_8	1342864	1343006	1	GGAAACCGAGTCAGGTTTTCGGCTGGCGGCGCTTAAATTTCAATTGCTGTCCT	ND	1
S43	CRISPR	S43_9	1769913	1770051	1	AGGTTTTCGGCTGGAGCGGCTTAAATTTCGATTGCTGTCCTCTTTCACA	ND	1
S45	CRISPR	S45_1	1213038	1213739	11	GTATTCCCCACGTACGTAGGGGTGATCC	-	4
S45	Cas cluster	CAS-TypeIE	1214110	1223562	8	cas3_TypeI, cse1_TypeIE, cse2_TypeIE, cas7_TypeIE, cas5_TypeIE, cas6_TypeIE, cas1_TypeIE, cas2_TypeIE		
S45	CRISPR	S45_2	1223592	1224291	11	GTATTCCCCACGTACGTAGGGGTGATCC	-	4
S45	CRISPR	S45_3	1225512	1226580	17	GTATTCCCCACGTACGTAGGGGTGATCC	-	4
S45	CRISPR	S45_4	1229544	1229754	3	GTATTCCCCACGTGTGTAGGGGTGATCC	-	1
S45	CRISPR	S45_5	1517544	1517686	1	GGAAACCGAGTCAGGTTTTCGGCTGGCGGCGCTTAAATTTCAATTGCTGTCCT	ND	1
S45	CRISPR	S45_6	1809956	1810094	1	TGTGAAAGAGGACAGCCATCGAAATTTAAGCCGCTCCAGCCGAAAACCT	ND	1
S45	CRISPR	S45_7	2191668	2191770	1	CCCCCTTATATGGCCGGAAGCTGC	ND	1
S45	CRISPR	S45_8	2232679	2233772	16	GTTTTAGAAGGATGTTAAATCAATAAGGTTAAACCC	ND	4
S47	CRISPR	S47_1	246812	246968	1	TGAACCCCTTGGTACAAGCGGACTTCTCAAAAAGAGA	ND	1
S47	CRISPR	S47_2	1149044	1149745	11	GTATTCCCCACGTACGTAGGGGTGATCC	-	4

S47	Cas cluster	CAS-TypeIE	1150116	1159568	8	cas3_TypeI, cse1_TypeIE, cse2_TypeIE, cas7_TypeIE, cas5_TypeIE, cas6_TypeIE, cas1_TypeIE, cas2_TypeIE		
S47	CRISPR	S47_3	1159598	1160297	11	GTATTCCCCACGTACGTAGGGGTGATCC	-	4
S47	CRISPR	S47_4	1161518	1162785	20	GTATTCCCCACGTACGTAGGGGTGATCC	-	4
S47	CRISPR	S47_5	1165749	1165959	3	GTATTCCCCACGTGTGTAGGGGTGATCC	-	1
S47	CRISPR	S47_6	1453749	1453891	1	GGAAACCGAGTCAGGTTTTCGGCTGGCGGCGCTTAAATTTCAATTGCTGTCCT	ND	1
S47	CRISPR	S47_7	1746161	1746285	1	TGTGAAAGAGGACAGCCATCGAAATTTAAGCCGCT	ND	1
S47	CRISPR	S47_8	2261155	2261257	1	CCCCCTTATATGGCCGGAAGCTGC	ND	1
S47	CRISPR	S47_9	2302166	2303259	16	GTTTTAGAAGGATGTTAAATCAATAAGGTTAAACCC	ND	4
S50	CRISPR	S50_1	56562	56664	1	CCCCCTTATATGGCCGGAAGCTGC	ND	1
S50	CRISPR	S50_2	335197	336025	12	GTTTTAGAAGGATGTTAAATCAATAAGGTTAAACCC	ND	4
S50	CRISPR	S50_3	364679	364835	1	TGAACCCCTTGGTACAAGCGGACTTCTCAAAAAGAGA	ND	1
S50	CRISPR	S50_4	704835	705045	3	GGATCACCCCTACACACGTGGGGAATAC	ND	1
S50	CRISPR	S50_5	708009	709078	17	GGATCACCCCTACGTACGTGGGGAATAC	ND	4
S50	CRISPR	S50_6	710299	710997	11	GGATCACCCCTACGTACGTGGGGAATAC	ND	4
S50	Cas cluster	CAS-TypeIE	711027	720479	8	cas2_TypeIE, cas1_TypeIE, cas6_TypeIE, cas5_TypeIE, cas7_TypeIE, cse2_TypeIE, cse1_TypeIE, cas3_TypeI		
S50	CRISPR	S50_7	720850	721551	11	GGATCACCCCTACGTACGTGGGGAATAC	ND	4
S50	CRISPR	S50_8	1056474	1056598	1	TGTGAAAGAGGACAGCCATCGAAATTTAAGCCGCT	ND	1
S50	CRISPR	S50_9	1095944	1096093	1	GTAAATAAAATTTACTTGTAAATAAAGTTTACTTGTAAATATTATTTAC	ND	1
S50	CRISPR	S50_10	1099024	1099390	5	GGGTTTAACCTTATTGATTTAACATCCTTCTAAAAC	-	3
S50	CRISPR	S50_11	1349323	1349465	1	AGGACAGCAATTGAAATTTAAGCGCCGCCAGCCGAAAACCTGACTCGGTTTCC	ND	1
S50	CRISPR	S50_12	1822771	1822892	1	ACTCGGTTTCCTGGTGTCTTACTTTTATTTGG	ND	1

S51	CRISPR	S51_1	798050	799801	26	GGGTTTAACCTTATTGATTTAACATCCTTCTAAAAC	-	4
S51	Cas cluster	CAS-TypeIIA	799830	806008	4	csn2_TypeIIA, cas2_TypeI-II-III, cas1_TypeII, cas9_TypeII		
S51	CRISPR	S51_2	823649	823777	1	CTTGCTGTCCGCTTTCACACGTTTGATCCAAATAACAGT	-	1
S51	CRISPR	S51_3	1733061	1733216	1	AACCCTTGCTACGAGCGGGATTTCGAAAATCGAAAG	ND	1
S53	CRISPR	S53_1	238668	239761	16	GGGTTTAACCTTATTGATTTAACATCCTTCTAAAAC	-	4
S53	CRISPR	S53_2	638588	639289	11	GTATTCCCCACGTACGTAGGGGTGATCC	-	4
S53	Cas cluster	CAS-TypeIE	639660	649112	8	cas3_TypeI, cse1_TypeIE, cse2_TypeIE, cas7_TypeIE, cas5_TypeIE, cas6_TypeIE, cas1_TypeIE, cas2_TypeIE		
S53	CRISPR	S53_3	649142	649841	11	GTATTCCCCACGTACGTAGGGGTGATCC	-	4
S53	CRISPR	S53_4	651062	652130	17	GTATTCCCCACGTACGTAGGGGTGATCC	-	4
S53	CRISPR	S53_5	655094	655304	3	GTATTCCCCACGTGTGTAGGGGTGATCC	-	1
S53	CRISPR	S53_6	966996	967145	1	GTAAATAAAATTTACTTGTAAATAAAGTTTACTTGTAAATATTATTTAC	ND	1
S53	CRISPR	S53_7	1391013	1391155	1	GGAAACCGAGTCAGGTTTTCGGCTGGCGGCGCTTAAATTTCAATTGCTGTCCT	ND	1
S53	CRISPR	S53_8	1683425	1683549	1	TGTGAAAGAGGACAGCCATCGAAATTTAAGCCGCT	ND	1
S53	CRISPR	S53_9	2462329	2462431	1	CCCCCTTATATGGCCGGAAGCTGC	ND	1
S58	CRISPR	S58_1	217068	218161	16	GTTTTAGAAGGATGTTAAATCAATAAGGTTAAACCC	ND	4
S58	CRISPR	S58_2	246843	246999	1	TGAACCCCTTGGTACAAGCGGACTTCTCAAAAAGAGA	ND	1
S58	CRISPR	S58_3	612104	612314	3	GGATCACCCCTACACACGTGGGGAATAC	ND	1
S58	CRISPR	S58_4	615278	616347	17	GGATCACCCCTACGTACGTGGGGAATAC	ND	4
S58	CRISPR	S58_5	617568	618266	11	GGATCACCCCTACGTACGTGGGGAATAC	ND	4
S58	Cas cluster	CAS-TypeIE	618296	627748	8	cas2_TypeIE, cas1_TypeIE, cas6_TypeIE, cas5_TypeIE, cas7_TypeIE, cse2_TypeIE, cse1_TypeIE, cas3_TypeI		

S58	CRISPR	S58_6	628119	628820	11	GGATCACCCCTACGTACGTGGGGAATAC	ND	4
S58	CRISPR	S58_7	1627562	1627704	1	AGGACAGCAATTGAAATTTAAGCGCCGCCAGCCGAAAACCTGACTCGGTTTCC	ND	1
S58	CRISPR	S58_8	1682854	1682992	1	TGTGAAAGAGGACAGCCATCGAAATTTAAGCCGCTCCAGCCGAAAACCT	ND	1
S58	CRISPR	S58_9	2462502	2462604	1	CCCCCTTATATGGCCGGAAGCTGC	ND	1
S59	CRISPR	S59_1	177123	178216	16	GGGTTTAACCTTATTGATTTAACATCCTTCTAAAAC	-	4
S59	CRISPR	S59_2	593248	593949	11	GTATTCCCCACGTACGTAGGGGTGATCC	-	4
S59	Cas cluster	CAS-TypeIE	594320	603772	8	cas3_TypeI, cse1_TypeIE, cse2_TypeIE, cas7_TypeIE, cas5_TypeIE, cas6_TypeIE, cas1_TypeIE, cas2_TypeIE		
S59	CRISPR	S59_3	603802	604501	11	GTATTCCCCACGTACGTAGGGGTGATCC	-	4
S59	CRISPR	S59_4	605722	606790	17	GTATTCCCCACGTACGTAGGGGTGATCC	-	4
S59	CRISPR	S59_5	609754	609964	3	GTATTCCCCACGTGTGTAGGGGTGATCC	-	1
S59	CRISPR	S59_6	1435507	1435649	1	GGAAACCGAGTCAGGTTTTCGGCTGGCGGCGCTTAAATTTCAATTGCTGTCCT	ND	1
S59	CRISPR	S59_7	1727919	1728057	1	TGTGAAAGAGGACAGCCATCGAAATTTAAGCCGCTCCAGCCGAAAACCT	ND	1
S59	CRISPR	S59_8	2506550	2506652	1	CCCCCTTATATGGCCGGAAGCTGC	ND	1
SG162	CRISPR	SG162_1	310104	310316	3	GTATTCCCCACGTACGTAGGGGTGATCCT	ND	1
SG162	CRISPR	SG162_2	310594	310745	2	GTATTCCCCACGTACGTAGGGGTGATCCT	ND	1
SG162	Cas cluster	CAS-TypeIE	311602	321054	8	cas3_TypeI, cse1_TypeIE, cse2_TypeIE, cas7_TypeIE, cas5_TypeIE, cas6_TypeIE, cas1_TypeIE, cas2_TypeIE		
SG162	CRISPR	SG162_3	321084	321174	1	GTATTCCCCACGTACGTAGGGGTGATCCT	ND	1
SG162	CRISPR	SG162_4	321572	321784	3	GTATTCCCCACGTACGTAGGGGTGATCCT	ND	1
SG162	CRISPR	SG162_5	323004	323216	3	GTATTCCCCACGTACGTAGGGGTGATCCT	ND	1
SG162	CRISPR	SG162_6	324103	324439	5	GTATTCCCCACGTACGTAGGGGTGATCCT	ND	4
SG162	CRISPR	SG162_7	327402	327612	3	GTATTCCCCACGTGTGTAGGGGTGATCC	-	1

SG162	CRISPR	SG162_8	449670	449812	1	AGGACAGCAATTGAAATTTAAGCGCCGCCAGCCGAAAACCTGACTCGGTTTCC	ND	1
SG162	CRISPR	SG162_9	1946477	1946579	1	CCCCCTTATATGGCCGGAAGCTGC	ND	1
SG162	Cas cluster	CAS-TypeIIA	1980174	1986353	4	cas9_TypeII, cas1_TypeII, cas2_TypeI-II-III, csn2_TypeIIA		
SG162	CRISPR	SG162_10	1986382	1986880	7	GTTTTAGAAGGATGTTAAATCAATAAGGTTAAACCC	ND	4
SG162	CRISPR	SG162_11	2474138	2474293	1	AAACCCTTGCTACGAGCGAAAGTCTAAAAAATAGAC	ND	1
