# Supplementary material for: Comparative genomics of Lentilactobacillus buchneri reveals strain-level hyperdiversity and broad-spectrum CRISPR immunity against human and livestock gut phages
Source: PLoS One. 2025 Jun 10;20(6):e0325832. doi: 10.1371/journal.pone.0325832 (PMC12151389; doi:10.1371/journal.pone.0325832)
Supplement: S2 Table — (PDF) [file pone.0325832.s002.pdf]

S2 Table. Putative prophages predicted in *L. buchneri* strains using PHASTEST

**Strain**	**Region**	**Start**	**Stop**	**Size (kb)**	**Completeness**	**Most commom phage**	**GC (%)**
177	1	552582	602799	50.217	intact	PHAGE_Lactob_Lb_NC_047983	39.19
177	2	2456958	2501769	44.811	intact	PHAGE_Lactob_T25_NC_048625	37.09
1012	2	125260	144345	19.085	incomplete	PHAGE_Lactob_phiAT3_NC_005893	41.35
1012	4	1724956	1757667	32.711	incomplete	PHAGE_Lactob_Lb_NC_047983	39.95
1012	1	60573	107330	46.757	intact	PHAGE_Lactob_T25_NC_048625	37.31
1012	5	2535118	2562571	27.453	intact	PHAGE_Lactob_Lj771_NC_010179	40.38
1014	1	941930	961435	19.505	intact	PHAGE_Lactob_T25_NC_048625	38.45
1014	2	1754111	1785835	31.724	intact	PHAGE_Lactob_LBR48_NC_027990	44.14
1014	3	2435369	2472979	37.61	intact	PHAGE_Lactob_Lb_NC_047983	38.77
ATCC_4005	1	141	19097	18.956	incomplete	PHAGE_Geobac_GBSV1_NC_008376	39.84
ATCC_4005	2	264615	289353	24.738	incomplete	PHAGE_Lactob_KC5a_NC_007924	43.48
ATCC_4005	3	282556	337904	55.348	intact	PHAGE_Lactob_T25_NC_048625	37.97
ATCC_4005	4	2472677	2509521	36.844	intact	PHAGE_Lister_B054_NC_009813	42.3
CD034	1	930146	984085	53.939	intact	PHAGE_Lactob_Lb_NC_047983	39.52
CD034	2	1547718	1592285	44.567	intact	PHAGE_Lactob_T25_NC_048625	37.62
CIRM-BIA_1514	1	94	13628	13.534	incomplete	PHAGE_Lactob_phig1e_NC_004305	40.02
CIRM-BIA_1514	2	275796	322156	46.36	intact	PHAGE_Lactob_T25_NC_048625	37.47
CIRM-BIA_1514	3	622944	656135	33.191	intact	PHAGE_Lactob_Lb_NC_047983	39.55
CIRM-BIA_1516	1	1267269	1307839	40.57	intact	PHAGE_Lactob_Lb_NC_047983	39.65
CIRM-BIA_1516	2	1570793	1619945	49.152	intact	PHAGE_Lactob_T25_NC_048625	37.35

CIRM-BIA_2081	1	65193	90663	25.47	intact	PHAGE_Lactob_T25_NC_048625	38.07
CIRM-BIA_2081	2	2005739	2060361	54.622	intact	PHAGE_Lactob_Lb_NC_047983	40.43
CIRM-BIA_2082	3	1596999	1617199	20.2	incomplete	PHAGE_Lactob_Lb_NC_047983	41.39
CIRM-BIA_2082	1	1153926	1179000	25.074	intact	PHAGE_Lactob_T25_NC_048625	38.22
CIRM-BIA_2083	1	505227	545206	39.979	intact	PHAGE_Lactob_T25_NC_048625	40.19
CIRM-BIA_2084	1	522690	565708	43.018	intact	PHAGE_Lactob_T25_NC_048625	37.54
CIRM-BIA_659	3	2471752	2478977	7.225	incomplete	PHAGE_Lactob_Lj771_NC_010179	40.52
CIRM-BIA_659	1	1987467	2043251	55.784	intact	PHAGE_Lactob_LBR48_NC_027990	42.57
CIRM-BIA_659	2	2270137	2336283	66.146	intact	PHAGE_Lactob_T25_NC_048625	39.52
CIRM-BIA_664	1	32468	39117	6.649	incomplete	PHAGE_Lactob_jlb1_NC_024206	39.31
CIRM-BIA_664	2	2119676	2175432	55.756	intact	PHAGE_Lactob_LBR48_NC_027990	42.57
CIRM-BIA_664	3	2440607	2484261	43.654	intact	PHAGE_Lactob_T25_NC_048625	37.96
CIRM-BIA_845	3	2464446	2473907	9.461	incomplete	PHAGE_Lactob_jlb1_NC_024206	39.86
CIRM-BIA_845	1	1982623	2038399	55.776	intact	PHAGE_Lactob_LBR48_NC_027990	42.57
CIRM-BIA_845	2	2291942	2336102	44.16	intact	PHAGE_Lactob_T25_NC_048625	38
DSM_20057	1	1010244	1064621	54.377	intact	PHAGE_Lactob_T25_NC_048625	39.7
DSM_20057	2	1054772	1111019	56.247	intact	PHAGE_Lactob_LBR48_NC_027990	40.39
FUA3252	1	912714	940204	27.49	intact	PHAGE_Lactob_T25_NC_048625	38.66
FUA3252	2	979969	1025532	45.563	intact	PHAGE_Lactob_Lb_NC_047983	39.76
LA1147	1	1631515	1673285	41.77	intact	PHAGE_Lactob_T25_NC_048625	37.67
LA1161B	1	43288	96021	52.733	intact	PHAGE_Lactob_Lj771_NC_010179	40.34

LA1161B	2	1678516	1708450	29.934	intact	PHAGE_Lactob_T25_NC_048625	38.38
LA1161C	1	52372	108359	55.987	intact	PHAGE_Lactob_jlb1_NC_024206	39.42
LA1161C	2	1631589	1671634	40.045	intact	PHAGE_Lactob_T25_NC_048625	38.05
LA1167	1	21836	98195	76.359	intact	PHAGE_Lactob_Lj771_NC_010179	39.47
LA1167	2	1663679	1693613	29.934	intact	PHAGE_Lactob_T25_NC_048625	38.38
LA1181	2	1662589	1670293	7.704	incomplete	PHAGE_Lactob_521B_NC_048752	37.27
LA1181	1	13095	60938	47.843	intact	PHAGE_Deep_s_D6E_NC_019544	40.09
LA1181	3	1707700	1739831	32.131	intact	PHAGE_Lactob_T25_NC_048625	37.92
LA1184	1	70484	122402	51.918	intact	PHAGE_Lactob_phig1e_NC_004305	41.35
LA1184	3	183694	244004	60.31	intact	PHAGE_Lactob_LBR48_NC_027990	40.87
LA1184	4	304205	334343	30.138	intact	PHAGE_Lactob_T25_NC_048625	38.67
MGB0786	2	2370519	2432993	62.474	intact	PHAGE_Lactob_LBR48_NC_027990	41.91
MGB0786	3	2465380	2500306	34.926	intact	PHAGE_Lactob_T25_NC_048625	37.74
MGB0786	4	2527222	2583541	56.319	intact	PHAGE_Lactob_Lj771_NC_010179	40.06
MGR2-32	1	2254500	2313435	58.935	intact	PHAGE_Lactob_Lb_NC_047983	40.11
MGR2-32	2	2495178	2520412	25.234	intact	PHAGE_Lactob_T25_NC_048625	38.34
NBRC_107764	1	1678810	1712843	34.033	intact	PHAGE_Lactob_T25_NC_048625	39.63
NBRC_107764	2	1759355	1825983	66.628	intact	PHAGE_Lactob_LBR48_NC_027990	42.09
NK01	2	2213042	2281023	67.981	intact	PHAGE_Lactob_LBR48_NC_027990	39.46
NK01	3	2382754	2408806	26.052	intact	PHAGE_Lactob_T25_NC_048625	38.12
NRRL_B-30929	1	1460070	1508182	48.112	intact	PHAGE_Lister_B054_NC_009813	41.27

NRRL_B-30929	2	1549566	1576362	26.796	intact	PHAGE_Lactob_T25_NC_048625	38.37
PC-C1	1	1284053	1331081	47.028	intact	PHAGE_Lactob_Lb_NC_047983	39.24
PC-C1	2	1518330	1565628	47.298	intact	PHAGE_Lactob_T25_NC_048625	38.07
S42	1	1003790	1029772	25.982	intact	PHAGE_Lactob_T25_NC_048625	37.77
S43	1	210204	237266	27.062	intact	PHAGE_Lactob_T25_NC_048625	37.76
S45	1	807112	834174	27.062	intact	PHAGE_Lactob_T25_NC_048625	37.76
S45	2	849195	879459	30.264	intact	PHAGE_Lactob_T25_NC_048625	37.65
S47	1	136767	162749	25.982	intact	PHAGE_Lactob_T25_NC_048625	37.77
S50	1	1363840	1431501	67.661	intact	PHAGE_Lactob_T25_NC_048625	37.27
S50	2	1431879	1457861	25.982	intact	PHAGE_Lactob_T25_NC_048625	37.77
S51	3	1819659	1831904	12.245	incomplete	PHAGE_Lactob_Sha1_NC_019489	38.61
S51	1	1490660	1514557	23.897	intact	PHAGE_Lactob_T25_NC_048625	36.96
S51	2	1774548	1805742	31.194	intact	PHAGE_Entero_vB_EfaS_AL2_NC_042127	40.81
S51	4	2450515	2506809	56.294	intact	PHAGE_Lactob_LBR48_NC_027990	41.57
S53	1	153022	179004	25.982	intact	PHAGE_Lactob_T25_NC_048625	37.77
S58	1	134099	161161	27.062	intact	PHAGE_Lactob_T25_NC_048625	37.76
S59	1	1333059	1360121	27.062	intact	PHAGE_Lactob_T25_NC_048625	37.76
S59	2	1375142	1405406	30.264	intact	PHAGE_Lactob_T25_NC_048625	37.65
SG162	1	1150562	1209524	58.962	intact	PHAGE_Lactob_T25_NC_048625	38.97
SG162	2	1725087	1772987	47.9	intact	PHAGE_Deep_s_D6E_NC_019544	40.15
SG162	3	2416388	2474823	58.435	intact	PHAGE_Lactob_Lb_NC_047983	39.82

1012	3	1208504	1232100	23.596	questionable	PHAGE_Coryne_Lederberg_NC_048790	43.05
CIRM-BIA_2082	2	1520421	1543565	23.144	questionable	PHAGE_Lactob_Lb_NC_047983	39.33
LA1175D	1	114643	140887	26.244	questionable	PHAGE_Clostr_phiCD6356_NC_015262	39.18
LA1184	2	123045	139802	16.757	questionable	PHAGE_Lactob_LBR48_NC_027990	40.06
MGB0786	1	424156	453291	29.135	questionable	PHAGE_Lactob_Sha1_NC_019489	38.7
NK01	1	664652	683944	19.292	questionable	PHAGE_Entero_LY0322_NC_042125	38.94
SG162	4	2629116	2645436	16.32	questionable	PHAGE_Paenib_Shelly_NC_041909	44.13
