# Supplementary material for: Comparative genomics of Lentilactobacillus buchneri reveals strain-level hyperdiversity and broad-spectrum CRISPR immunity against human and livestock gut phages
Source: PLoS One. 2025 Jun 10;20(6):e0325832. doi: 10.1371/journal.pone.0325832 (PMC12151389; doi:10.1371/journal.pone.0325832)
Supplement: S3 Table — (PDF) [file pone.0325832.s003.pdf]

S3 Table. Putative plasmids found in *L. buchneri* strains

Strain	Identity	NCBI Accession	GC (%)	Length
1014	99.88%	NZ_CP064865.1	37.52%	3699
1014	99.18%	NZ_CP012658.1	43.39%	5603
ATCC_4005	100.00%	NZ_CP073067.1	41.57%	28633
CD034	100.00%	NC_018611.1	37.75%	56473
CD034	100.00%	NC_016035.1	38.35%	3424
CD034	100.00%	NC_016034.1	38.60%	2707
CIRM-BIA_2082	99.93%	NC_016035.1	38.35%	3424
CIRM-BIA_659	99.83%	NZ_CP073067.1	41.57%	28633
CIRM-BIA_664	99.87%	NZ_CP073067.1	41.57%	28633
CIRM-BIA_845	99.87%	NZ_CP073067.1	41.57%	28633
DSM_20057	99.83%	NZ_CP073067.1	41.57%	28633
LA1147	99.75%	NC_019219.1	42.41%	5529
LA1161B	99.01%	NZ_CP065817.1	45.06%	2745
LA1167	99.01%	NZ_CP065817.1	45.06%	2745
LA1175D	99.45%	NZ_CP065817.1	45.06%	2745
LA1175D	99.29%	LR962096.1	36.34%	809
LA1181	99.94%	NZ_CP043613.1	41.21%	64413
LA1181	99.46%	NZ_CP065817.1	45.06%	2745
LA1181	99.29%	LR962096.1	36.34%	809
LA1181	99.02%	NC_012220.1	37.78%	2112
MGB0786	100.00%	NZ_CP043616.1	40.67%	39778
MGB0786	100.00%	NZ_CP043614.1	37.41%	14316
MGB0786	100.00%	NZ_CP043613.1	41.21%	64413
NBRC_107764	99.23%	NZ_CP073067.1	41.57%	28633
NK01	99.89%	NZ_CP063752.1	37.41%	1815

NK01	99.85%	NZ_MT436440.1	38.72%	1356
NK01	99.85%	NC_012548.1	37.25%	1815
NK01	99.79%	NC_021574.1	37.70%	1788
NK01	99.23%	NC_016635.1	37.58%	1815
NRRL_B-30929	100.00%	NC_015420.1	38.09%	52697
NRRL_B-30929	100.00%	NC_015429.1	40.41%	18513
NRRL_B-30929	100.00%	NC_015421.1	37.63%	10798
