# Supplementary material for: Comparative genomics of Lentilactobacillus buchneri reveals strain-level hyperdiversity and broad-spectrum CRISPR immunity against human and livestock gut phages
Source: PLoS One. 2025 Jun 10;20(6):e0325832. doi: 10.1371/journal.pone.0325832 (PMC12151389; doi:10.1371/journal.pone.0325832)
Supplement: S4 Table — (PDF) [file pone.0325832.s004.pdf]

S4 Table. Megablast search results showing best CDS hits targeted by spacers.

spacer_name	sequence	length	accession	blast_hit	CDS	Score	Expect	Identities	Gaps	Strand	blastp_result
1012.1_spacer9	AAAGTAATTCCAAATATGCTAATGAATTTG	30	CP012024	Bacillus smithii strain DSM 4216, complete genome	DNA double-strand break repair Rad50 ATPase	26.3 bits(13)	0,73	13/13(100%)	0/13(0%)	Plus/Minus	
1012.2_spacer6	TTAATGAAACAGATAAATCGGTCAATGACT	30	BK046275	MAG TPA_asm: Caudoviricetes sp. isolate ctRj51, partial genome	hypothetical protein	44.1 bits(22)	3,00E-06	28/30(93%)	0/30(0%)	Plus/Plus	
1012.1_spacer6	TTAATGAAACAGATAAATCGGTCAATGACT	30	BK046275	MAG TPA_asm: Caudoviricetes sp. isolate ctRj51, partial genome	hypothetical protein	44.1 bits(22)	3,00E-06	28/30(93%)	0/30(0%)	Plus/Plus	
RUG14303.1_spacer2	CATCAGTTAGTTGATTATTATTCATGCTGG	30	CP086132	Lacticaseibacillus paracasei strain BCRC-16100 chromosome	SEC10/PgrA surface exclusion domain-containing protein	44.1 bits(22)	0,24	22/22(100%)	0/22(0%)	Plus/Minus	

CIRM-BIA_2084.1_spacer1	CCACTTACTTGCCGTTAAAGCGGGATATCG	30	CP053342	Lactiplantibacillus paraplantarum strain CK401 plasmid pCK401E, complete sequence	transposase for insertion sequence element IS905	54.0 bits(27)	2,00E-08	27/27(100%)	0/27(0%)	Plus/Plus	
CIRM-BIA_2083.1_spacer37	CCACTTACTTGCCGTTAAAGCGGGATATCG	30	CP053342	Lactiplantibacillus paraplantarum strain CK401 plasmid pCK401E, complete sequence	transposase for insertion sequence element IS905	54.0 bits(27)	2,00E-08	27/27(100%)	0/27(0%)	Plus/Plus	
LA1181.1_spacer26	GCTCGTTAATATAAGCCGCAATATCCGTTT	30	CP141334	Lactiplantibacillus plantarum strain CYLB101 plasmid pCYLB101-3, complete sequence	MobV family relaxase	60.0 bits(30)	4,00E-06	30/30(100%)	0/30(0%)	Plus/Plus	

NRRL_B-30929.1_spacer39	GTAGCTACTTTTAAGATCACGAAACTCGGT	30	CP026508	Lactiplantibacillus plantarum strain NCIMB 700965.EF.A plasmid punamed3, complete sequence	replication initiation protein	60.0 bits(30)	1,00E-08	30/30(100%)	0/30(0%)	Plus/Plus	
LA1161B.1_spacer39	CAATGATTTGAAGGATTTAAAAAAACTAGT	30	CP003043	Lactobacillus buchneri CD034, complete genome	hypothetical protein	60.0 bits(30)	4,00E-06	30/30(100%)	0/30(0%)	Plus/Plus	hypothetical protein [Lentilactobacillus]
LA1161C.1_spacer39	CAATGATTTGAAGGATTTAAAAAAACTAGT	30	CP003043	Lactobacillus buchneri CD034, complete genome	hypothetical protein	60.0 bits(30)	4,00E-06	30/30(100%)	0/30(0%)	Plus/Plus	hypothetical protein [Lentilactobacillus]
LA1167.2_spacer39	CAATGATTTGAAGGATTTAAAAAAACTAGT	30	CP003043	Lactobacillus buchneri CD034, complete genome	hypothetical protein	60.0 bits(30)	4,00E-06	30/30(100%)	0/30(0%)	Plus/Plus	hypothetical protein [Lentilactobacillus]
CIRM-BIA_2082.1_spacer20	CCAACGTTGATGAGAACCCGGGCGAATACC	30	CP002652	Lactobacillus buchneri NRRL B-	Protein of unknown functio	44.1 bits(	2,00E-05	28/30(93%)	0/30(0%)	Plus/Minus	

				30929, complete genome	n DUF2213	22)					
CIRM-BIA_2083.1_spacer20	CCAACGTTGATGAGAACCCGGGCGAATACC	30	CP002652	Lactobacillus buchneri NRRL B-30929, complete genome	Protein of unknown function DUF2213	44.1 bits(22)	2,00E-05	28/30(93%)	0/30(0%)	Plus/Minus	
NRRL_B-30929.1_spacer14	CCAACGTTGATGAGAACCCGGGCGAATACC	30	CP002652	Lactobacillus buchneri NRRL B-30929, complete genome	Protein of unknown function DUF2213	44.1 bits(22)	2,00E-05	28/30(93%)	0/30(0%)	Plus/Minus	
LA1147.1_spacer20	CCAACGTTGATGAGAACCCGGGCGAATACC	30	CP002652	Lactobacillus buchneri NRRL B-30929, complete genome	Protein of unknown function DUF2213	44.1 bits(22)	2,00E-05	28/30(93%)	0/30(0%)	Plus/Minus	
LA1161B.1_spacer20	CCAACGTTGATGAGAACCCGGGCGAATACC	30	CP002652	Lactobacillus buchneri NRRL B-30929, complete genome	Protein of unknown function DUF2213	44.1 bits(22)	2,00E-05	28/30(93%)	0/30(0%)	Plus/Minus	
LA1161C.1_spacer20	CCAACGTTGATGAGAACCCGGGCGAATACC	30	CP002652	Lactobacillus buchneri NRRL	Protein of unknown	44.1 bits(	2,00E-05	28/30(93%)	0/30(0%)	Plus/Minus	

				B-30929, complete genome	function DUF2213	22)					
LA1167.2_spacer20	CCAACGTTGATGAGAACCCGGGCGAATACC	30	CP002652	Lactobacillus buchneri NRRL B-30929, complete genome	Protein of unknown function DUF2213	44.1 bits(22)	2,00E-05	28/30(93%)	0/30(0%)	Plus/Minus	
LA1175D.1_spacer20	CCAACGTTGATGAGAACCCGGGCGAATACC	30	CP002652	Lactobacillus buchneri NRRL B-30929, complete genome	Protein of unknown function DUF2213	44.1 bits(22)	2,00E-05	28/30(93%)	0/30(0%)	Plus/Minus	
S51.1_spacer20	CCAACGTTGATGAGAACCCGGGCGAATACC	30	CP002652	Lactobacillus buchneri NRRL B-30929, complete genome	Protein of unknown function DUF2213	44.1 bits(22)	2,00E-05	28/30(93%)	0/30(0%)	Plus/Minus	
CIRM-BIA_2084.1_spacer9	CCAACGTTGATGAGAACCCGGGCGAATACC	30	CP002652	Lactobacillus buchneri NRRL B-30929, complete genome	Protein of unknown function DUF2213	44.1 bits(22)	2,00E-05	28/30(93%)	0/30(0%)	Plus/Minus	
LA1161B.1_spacer40	GTATTGAAGCAAGCTTTTAGACTCATCTAG	30	NC_048753	Lactobacillus phage	Mg2+/Co2+ transpo	52.0 bit	6,00	29/30(97%)	0/30(0%)	Plus/Minus	

				3-521, complete genome	rt protein	s(26)	E-10				
LA1161C.1_spacer40	GTATTGAAGCAAGCTTTTAGACTCATCTAG	30	NC_048753	Lactobacillus phage 3-521, complete genome	Mg2+/Co2+ transport protein	52.0 bits(26)	6,00E-10	29/30(97%)	0/30(0%)	Plus/Minus	
LA1167.2_spacer40	GTATTGAAGCAAGCTTTTAGACTCATCTAG	30	NC_048753	Lactobacillus phage 3-521, complete genome	Mg2+/Co2+ transport protein	52.0 bits(26)	6,00E-10	29/30(97%)	0/30(0%)	Plus/Minus	
RUG14303.1_spacer3	CTCCCATTAATCTAGTTGATGATGTGAACT	30	NC_047983	Lactobacillus phage Lb, complete genome	tail length tape measure protein	52.0 bits(26)	0,001	29/30(97%)	0/30(0%)	Plus/Plus	
LA1181.1_spacer22	CCTTGCACAAAGCGGCCGCCACCTTTTTCC	30	CP025208	Latilactobacillus sakei strain WiKim0074 plasmid pLSW74_2, complete sequence	conjugal transfer protein	60.0 bits(30)	1,00E-09	30/30(100%)	0/30(0%)	Plus/Plus	

MGB0786.1_spacer22	CCTTGCACAAAGCGGCCGCCACCTTTTTCC	30	CP025208	Latilactobacillus sakei strain WiKim0074 plasmid pLSW74_2, complete sequence	conjugal transfer protein	60.0 bits(30)	1,00E-09	30/30(100%)	0/30(0%)	Plus/Plus	
NRRL_B-30929.1_spacer22	CCTTGCACAAAGCGGCCGCCACCTTTTTCC	30	CP025208	Latilactobacillus sakei strain WiKim0074 plasmid pLSW74_2, complete sequence	conjugal transfer protein	60.0 bits(30)	1,00E-09	30/30(100%)	0/30(0%)	Plus/Plus	
PC-C1.1_spacer22	CCTTGCACAAAGCGGCCGCCACCTTTTTCC	30	CP025208	Latilactobacillus sakei strain WiKim0074 plasmid pLSW74_2, complete sequence	conjugal transfer protein	60.0 bits(30)	1,00E-09	30/30(100%)	0/30(0%)	Plus/Plus	
LA1147.1_spacer23	AATGGCCGTTGCGGAGCGCTTTTTTTGTTG	30	CP073066	Lentilactobacillus buchneri strain ATCC	PTS sugar transporter subunit IIC	24.3 bits(12)	17	12/12(100%)	0/12(0%)	Plus/Minus	

				4005 chromosome, complete genome							
LA1161B.1_spacer23	AATGGCCGTTGCGGAGCGCTTTTTTTGTTG	30	CP073066	Lentilactobacillus buchneri strain ATCC 4005 chromosome, complete genome	PTS sugar transporter subunit IIC	24.3 bits(12)	17	12/12(100%)	0/12(0%)	Plus/Minus	
LA1161C.1_spacer23	AATGGCCGTTGCGGAGCGCTTTTTTTGTTG	30	CP073066	Lentilactobacillus buchneri strain ATCC 4005 chromosome, complete genome	PTS sugar transporter subunit IIC	24.3 bits(12)	17	12/12(100%)	0/12(0%)	Plus/Minus	
LA1167.2_spacer23	AATGGCCGTTGCGGAGCGCTTTTTTTGTTG	30	CP073066	Lentilactobacillus buchneri strain ATCC 4005 chromosome, complete genome	PTS sugar transporter subunit IIC	24.3 bits(12)	17	12/12(100%)	0/12(0%)	Plus/Minus	

LA1167.2_spacer23	AATGGCCGTTGCGGAGCGCTTTTTTTGTTG	30	CP073066	Lentilactobacillus buchneri strain ATCC 4005 chromosome, complete genome	PTS sugar transporter subunit IIC	24.3 bits(12)	17	12/12(100%)	0/12(0%)	Plus/Minus	
FUA3252.1_spacer9	AATGGCCGTTGCGGAGCGCTTTTTTTGTTG	30	CP073066	Lentilactobacillus buchneri strain ATCC 4005 chromosome, complete genome	PTS sugar transporter subunit IIC	24.3 bits(12)	17	12/12(100%)	0/12(0%)	Plus/Plus	
ATCC_4005.1_spacer6	TTTCCGTCGGCAATAGCTTGCTGAGTTTCC	30	CP073066	Lentilactobacillus buchneri strain ATCC 4005 chromosome, complete genome	low molecular weight phosphotyrosine protein phosphatase	26.3 bits(13)	4,3	13/13(100%)	0/13(0%)	Plus/Plus	
CIRM-BIA_659.1_spacer6	TTTCCGTCGGCAATAGCTTGCTGAGTTTCC	30	CP073066	Lentilactobacillus buchneri strain ATCC 4005 chromosome,	low molecular weight phosphotyrosine protein	26.3 bits(13)	4,3	13/13(100%)	0/13(0%)	Plus/Plus	

				complete genome	phosphatase						
CIRM-BIA_664.1_spacer6	TTTCCGTCGGCAATAGCTTGCTGAGTTTCC	30	CP073066	Lentilactobacillus buchneri strain ATCC 4005 chromosome, complete genome	low molecular weight phosphotyrosine protein phosphatase	26.3 bits(13)	4, 3	13/13(100%)	0/13(0%)	Plus/Plus	
CIRM-BIA_845.1_spacer6	TTTCCGTCGGCAATAGCTTGCTGAGTTTCC	30	CP073066	Lentilactobacillus buchneri strain ATCC 4005 chromosome, complete genome	low molecular weight phosphotyrosine protein phosphatase	26.3 bits(13)	4, 3	13/13(100%)	0/13(0%)	Plus/Plus	
DSM_20057.1_spacer6	TTTCCGTCGGCAATAGCTTGCTGAGTTTCC	30	CP073066	Lentilactobacillus buchneri strain ATCC 4005 chromosome, complete genome	low molecular weight phosphotyrosine protein phosphatase	26.3 bits(13)	4, 3	13/13(100%)	0/13(0%)	Plus/Plus	

LA1181.1_spacer27	GAGATGTTGTGACGGCCAGCTTTATTTCTA	30	AP028329	Lentilactobacillus buchneri subsp. silagei MGR2-32 DNA, complete genome	hypothetical protein	60.0 bits(30)	4,00E-06	30/30(100%)	0/30(0%)	Plus/Plus	DUF2213 domain-containing protein [Lentilactobacillus]
LA1184.1_spacer1	TGCGTACCGATGAAACCACACCGCAAATTG	30	AP028329	Lentilactobacillus buchneri subsp. silagei MGR2-32 DNA, complete genome	hypothetical protein	44.1 bits(22)	0,24	28/30(93%)	0/30(0%)	Plus/Minus	**hypothetical protein [Lentilactobacillus buchneri]**
NRRL_B-30929.1_spacer40	TATCGAAACCGTTATTAACGGCCATCATGT	30	CP047122	Lentilactobacillus hilgardii strain FLUB plasmid unnamed1	MobA/MobL family	22.3 bits(11)	27	11/11(100%)	0/11(0%)	Plus/Minus	
LA1161B.1_spacer34	CATGGTGCTTCAAATGAGGCTGCTTATTTC	30	CP050262	Lentilactobacillus hilgardii strain LMG 07934 chromosome, complete genome	hypothetical protein	60.0 bits(30)	4,00E-06	30/30(100%)	0/30(0%)	Plus/Plus	phage tail tip lysozyme [Lentilactobacillus hilgardii]

LA1161C.1_spacer34	CATGGTGCTTCAAATGAGGCTGCTTATTTC	30	CP050262	Lentilactobacillus hilgardii strain LMG 07934 chromosome, complete genome	hypothetical protein	60.0 bits(30)	4,00E-06	30/30(100%)	0/30(0%)	Plus/Plus	phage tail tip lysozyme [Lentilactobacillus hilgardii]
LA1167.2_spacer34	CATGGTGCTTCAAATGAGGCTGCTTATTTC	30	CP050262	Lentilactobacillus hilgardii strain LMG 07934 chromosome, complete genome	hypothetical protein	60.0 bits(30)	4,00E-06	30/30(100%)	0/30(0%)	Plus/Plus	phage tail tip lysozyme [Lentilactobacillus hilgardii]
LA1161B.1_spacer25	CTAGTTCAGCGTGTGAGACACCTGAACCTT	30	CP050262	Lentilactobacillus hilgardii strain LMG 07934 chromosome, complete genome	hypothetical protein	48.1 bits(24)	0,015	27/28(96%)	0/28(0%)	Plus/Minus	phage tail tip lysozyme [Lentilactobacillus hilgardii]
LA1161C.1_spacer25	CTAGTTCAGCGTGTGAGACACCTGAACCTT	30	CP050262	Lentilactobacillus hilgardii strain LMG 07934 chromosome,	hypothetical protein	48.1 bits(24)	0,015	27/28(96%)	0/28(0%)	Plus/Minus	phage tail tip lysozyme [Lentilactobacillus hilgardii]

				complete genome							
LA1167.2_spacer25	CTAGTTCAGCGTGTGAGACACCTGAACCTT	30	CP050262	Lentilactobacillus hilgardii strain LMG 07934 chromosome, complete genome	hypothetical protein	48.1 bits(24)	0,015	27/28(96%)	0/28(0%)	Plus/Minus	phage tail tip lysozyme [Lentilactobacillus hilgardii]
LA1181.1_spacer16	ACGGCCCCAACTTGCTTAACGCCAGAAAGA	30	CP018796	Lentilactobacillus parabuchneri strain FAM21731 chromosome, complete genome	Baseplate J-like protein	44.1 bits(22)	0,24	28/30(93%)	0/30(0%)	Plus/Minus	
MGB0786.1_spacer16	ACGGCCCCAACTTGCTTAACGCCAGAAAGA	30	CP018796	Lentilactobacillus parabuchneri strain FAM21731 chromosome, complete genome	Baseplate J-like protein	44.1 bits(22)	0,24	28/30(93%)	0/30(0%)	Plus/Minus	

NRRL_B-30929.1_spacer16	ACGGCCCCAACTTGCTTAACGCCAGAAAGA	30	CP018796	Lentilactobacillus parabuchneri strain FAM21731 chromosome, complete genome	Baseplate J-like protein	44.1 bits(22)	0, 24	28/30(93%)	0/30(0%)	Plus/Minus	
LA1184.1_spacer5	CCGATTCAAGTTGCCAATCCCGATACTCAT	30	CP018796	Lentilactobacillus parabuchneri strain FAM21731 chromosome, complete genome	hypothetical protein	52.0 bits(26)	0, 001	26/26(100%)	0/26(0%)	Plus/Minus	phage tail tip lysozyme [Lentilactobacillus buchneri]
LA1184.1_spacer7	CCTTTAACCTGCGGATTAATCAATCGAGTA	30	CP018796	Lentilactobacillus parabuchneri strain FAM21731 chromosome, complete genome	hypothetical protein	60.0 bits(30)	4, 00E-06	30/30(100%)	0/30(0%)	Plus/Minus	phage tail tip lysozyme [Lentilactobacillus buchneri]
NRRL_B-30929.1_spacer41	CTCCTTTATTTAGTAGATTCATTTAAGTGA	30	CP018796	Lentilactobacillus parabuchneri strain	hypothetical protein	60.0 bits(30)	4, 00E-06	30/30(100%)	0/30(0%)	Plus/Minus	phage tail tip lysozyme [Lentilactobacil

		30		FAM21731 chromosome, complete genome							lus buchneri]
LA1184.1_spacer14	TCTTTCTGGCGTTAAGCAAGTTGGGGCCGT	30	CP018796	Lentilactobacillus parabuchneri strain FAM21731 chromosome, complete genome	Baseplate J-like protein	60.0 bits(30)	4,00E-06	30/30(100%)	0/30(0%)	Plus/Plus	
CIRM-BIA_2084.1_spacer10	TAATTCTGTCGATTATCTTTAGCTGCGTTA	30	BK020950	MAG TPA_asm: Caudoviricetes sp. isolate ctjNK7, partial genome	AAA domain protein	52.0 bits(26)	0,001	29/30(97%)	0/30(0%)	Plus/Plus	
1012.1_spacer10	TAATTCTGTCGATTATCTTTAGCTGCGTTA	30	BK020950	MAG TPA_asm: Caudoviricetes sp. isolate ctjNK7, partial genome	AAA domain protein	52.0 bits(26)	0,001	29/30(97%)	0/30(0%)	Plus/Plus	
CIRM-BIA_2082.1_spacer19	TAATTCTGTCGATTATCTTTAGCTGCGTTA	30	BK020950	MAG TPA_asm: Caudovi	AAA domain protein	52.0 bits(	0,001	29/30(97%)	0/30(0%)	Plus/Plus	

				ricetes sp. isolate ctjNK7, partial genome		26)					
CIRM-BIA_2083.1_spacer19	TAATTCTGTCGATTATCTTTAGCTGCGTTA	30	BK020950	MAG TPA_asm: Caudoviricetes sp. isolate ctjNK7, partial genome	AAA domain protein	52.0 bits(26)	0,001	29/30(97%)	0/30(0%)	Plus/Plus	
LA1161B.1_spacer19	TAATTCTGTCGATTATCTTTAGCTGCGTTA	30	BK020950	MAG TPA_asm: Caudoviricetes sp. isolate ctjNK7, partial genome	AAA domain protein	52.0 bits(26)	0,001	29/30(97%)	0/30(0%)	Plus/Plus	
LA1161C.1_spacer19	TAATTCTGTCGATTATCTTTAGCTGCGTTA	30	BK020950	MAG TPA_asm: Caudoviricetes sp. isolate ctjNK7, partial genome	AAA domain protein	52.0 bits(26)	0,001	29/30(97%)	0/30(0%)	Plus/Plus	
LA1167.2_spacer19	TAATTCTGTCGATTATCTTTAGCTGCGTTA	30	BK020950	MAG TPA_asm: Caudoviricetes sp. isolate ctjNK7,	AAA domain protein	52.0 bits(26)	0,001	29/30(97%)	0/30(0%)	Plus/Plus	

				partial genome							
S51.1_spacer19	TAATTCTGTCGATTATCTTTAGCTGCGTTA	30	BK020950	MAG TPA_asm: Caudoviricetes sp. isolate ctjNK7, partial genome	AAA domain protein	52.0 bits(26)	0,001	29/30(97%)	0/30(0%)	Plus/Plus	
LA1147.1_spacer24	CGTTGACTGGTTTGTATAGCCACCACTCAC	30	BK046275	MAG TPA_asm: Caudoviricetes sp. isolate ctRj51, partial genome	portal protein	52.0 bits(26)	3,00E-06	29/30(97%)	0/30(0%)	Plus/Minus	
LA1175D.1_spacer24	CGTTGACTGGTTTGTATAGCCACCACTCAC	30	BK046275	MAG TPA_asm: Caudoviricetes sp. isolate ctRj51, partial genome	portal protein	52.0 bits(26)	3,00E-06	29/30(97%)	0/30(0%)	Plus/Minus	
1012.2_spacer3	ATGCTTCGCCAACTTATCCATATATCGTTT	30	BK038207	MAG TPA_asm: Caudoviricetes sp. isolate ctXyb2, partial genome	tail completion protein	60.0 bits(30)	4,00E-06	30/30(100%)	0/30(0%)	Plus/Plus	

SG162.2_spacer1	CTTGCCGATCCACAACCGATGTAAATTCATCA	32	BK021565	MAG TPA_asm: Caudoviricetes sp. isolate ctZol16, partial genome	hypothetical protein	48.2 bits(52)	3,00E-05	29/31(94%)	0/31(0%)	Plus/Minus	GNAT family N-acetyltransferase [Bacteroidaceae bacterium] / ABC transporter ATP-binding protein [Ruminiclostridium sp.]
177.4_spacer1	CTTGCCGATCCACAACCGATGTAAATTCATCAA	33	BK021565	MAG TPA_asm: Caudoviricetes sp. isolate ctZol16, partial genome	hypothetical protein	50.0 bits(54)	7,00E-06	30/32(94%)	0/32(0%)	Plus/Minus	GNAT family N-acetyltransferase [Bacteroidaceae bacterium] / ABC transporter ATP-binding protein [Ruminiclostridium sp.]

CD034.3_spacer1	CTTGCCGATCCACAACCGATGTAAATTCATCAA	33	BK021565	MAG TPA_asm: Caudoviricetes sp. isolate ctZol16, partial genome	hypothetical protein	50.0 bits(54)	7,00E-06	30/32(94%)	0/32(0%)	Plus/Minus	GNAT family N-acetyltransferase [Bacteroidaceae bacterium] / ABC transporter ATP-binding protein [Ruminiclostridium sp.]
CIRM-BIA_1514.3_spacer1	CTTGCCGATCCACAACCGATGTAAATTCATCAA	33	BK021565	MAG TPA_asm: Caudoviricetes sp. isolate ctZol16, partial genome	hypothetical protein	50.0 bits(54)	7,00E-06	30/32(94%)	0/32(0%)	Plus/Minus	GNAT family N-acetyltransferase [Bacteroidaceae bacterium] / ABC transporter ATP-binding protein [Ruminiclostridium sp.]

MGR2-32.4_spacer1	CTTGCCGATCCACAACCGATGTAAATTCATCAA	33	BK021565	MAG TPA_asm: Caudoviricetes sp. isolate ctZol16, partial genome	hypothetical protein	50.0 bits(54)	7,00E-06	30/32(94%)	0/32(0%)	Plus/Minus	GNAT family N-acetyltransferase [Bacteroidaceae bacterium] / ABC transporter ATP-binding protein [Ruminiclostridium sp.]
S42.4_spacer1	CTTGCCGATCCACAACCGATGTAAATTCATCAA	33	BK021565	MAG TPA_asm: Caudoviricetes sp. isolate ctZol16, partial genome	hypothetical protein	50.0 bits(54)	7,00E-06	30/32(94%)	0/32(0%)	Plus/Minus	GNAT family N-acetyltransferase [Bacteroidaceae bacterium] / ABC transporter ATP-binding protein [Ruminiclostridium sp.]

S43.4_spacer1	CTTGCCGATCCACAACCGATGTAAATTCATCAA	33	BK021565	MAG TPA_asm: Caudoviricetes sp. isolate ctZoI16, partial genome	hypothetical protein	50.0 bits(54)	7,00E-06	30/32(94%)	0/32(0%)	Plus/Minus	GNAT family N-acetyltransferase [Bacteroidaceae bacterium] / ABC transporter ATP-binding protein [Ruminiclostridium sp.]
S45.3_spacer1	CTTGCCGATCCACAACCGATGTAAATTCATCAA	33	BK021565	MAG TPA_asm: Caudoviricetes sp. isolate ctZoI16, partial genome	hypothetical protein	50.0 bits(54)	7,00E-06	30/32(94%)	0/32(0%)	Plus/Minus	GNAT family N-acetyltransferase [Bacteroidaceae bacterium] / ABC transporter ATP-binding protein [Ruminiclostridium sp.]

S53.4_spacer1	CTTGCCGATCCACAACCGATGTAAATTCATCAA	33	BK021565	MAG TPA_asm: Caudoviricetes sp. isolate ctZol16, partial genome	hypothetical protein	50.0 bits(54)	7,00E-06	30/32(94%)	0/32(0%)	Plus/Minus	GNAT family N-acetyltransferase [Bacteroidaceae bacterium] / ABC transporter ATP-binding protein [Ruminiclostridium sp.]
S59.4_spacer1	CTTGCCGATCCACAACCGATGTAAATTCATCAA	33	BK021565	MAG TPA_asm: Caudoviricetes sp. isolate ctZol16, partial genome	hypothetical protein	50.0 bits(54)	7,00E-06	30/32(94%)	0/32(0%)	Plus/Minus	GNAT family N-acetyltransferase [Bacteroidaceae bacterium] / ABC transporter ATP-binding protein [Ruminiclostridium sp.]

CIRM-BIA_1516.2_spacer25	CTTGCCGATCCACAACCGATGTAAATTCATCAA	33	BK021565	MAG TPA_asm: Caudoviricetes sp. isolate ctZol16, partial genome	hypothetical protein	50.0 bits(54)	7,00E-06	30/32(94%)	0/32(0%)	Plus/Minus	GNAT family N-acetyltransferase [Bacteroidaceae bacterium] / ABC transporter ATP-binding protein [Ruminiclostridium sp.]
CIRM-BIA_2081.2_spacer25	CTTGCCGATCCACAACCGATGTAAATTCATCAA	33	BK021565	MAG TPA_asm: Caudoviricetes sp. isolate ctZol16, partial genome	hypothetical protein	50.0 bits(54)	7,00E-06	30/32(94%)	0/32(0%)	Plus/Minus	GNAT family N-acetyltransferase [Bacteroidaceae bacterium] / ABC transporter ATP-binding protein [Ruminiclostridium sp.]

S50.2_spacer25	CTTGCCGATCCACAACCGATGTAAATTCATCAA	33	BK021565	MAG TPA_asm: Caudoviricetes sp. isolate ctZol16, partial genome	hypothetical protein	50.0 bits(54)	7,00E-06	30/32(94%)	0/32(0%)	Plus/Minus	GNAT family N-acetyltransferase [Bacteroidaceae bacterium] / ABC transporter ATP-binding protein [Ruminiclostridium sp.]
S58.2_spacer25	CTTGCCGATCCACAACCGATGTAAATTCATCAA	33	BK021565	MAG TPA_asm: Caudoviricetes sp. isolate ctZol16, partial genome	hypothetical protein	50.0 bits(54)	7,00E-06	30/32(94%)	0/32(0%)	Plus/Minus	GNAT family N-acetyltransferase [Bacteroidaceae bacterium] / ABC transporter ATP-binding protein [Ruminiclostridium sp.]
LA1184.1_spacer4	CTAATCGCGATTTTATCGGCATGGAGTTAG	30	CP042382	Pistricoccus aurantiacus strain	sulfite oxidase	42.1 bits(	1,00E-05	21/21(100%)	0/21(0%)	Plus/Plus	

				CBA4606 chromosome, complete genome		21)					
LA1184.1_spacer2	CGGCAGGAGGTGTTGAAAATGGGTACTAAC	30	XM_066173474	PREDICTED: Pocillopora verrucosa EH domain-binding protein 1-like protein 1 (LOC136283913), mRNA	EH domain-binding protein 1-like protein 1	40.1 bits(20)	1, 9	20/20(100%)	0/20(0%)	Plus/Plus	
LA1184.1_spacer3	AATATTACACTGCTAAGGGTCTGTATGAAG	30	XM_017682291.2	Pygocentrus nattereri ArfGAP with SH3 domain, ankyrin repeat and PH domain 2b (asap2b), mRNA	arf-GAP with SH3	38.2 bits(19)	7, 7	19/19(100%)	0/19(0%)	Plus/Minus	
LA1184.1_spacer6	TTACGGAGCCAATAGTTTTAGTTTGCGGCG	30	XM_023461217	Trichogramma pretiosum midasin-like (LOC10	midasin-like	42.1 bits(21)	2,00E-04	21/21(100%)	0/21(0%)	Plus/Plus	

				6657303), mRNA							
NBRC_107764.1_spacer7	GGAAACTCAGCAAGCTATTGCCGACGGAAA	30	XM_001330005.1	Trichomonas vaginalis G3 uncharacterized protein (TVAG G3_0233020), partial mRNA	hypothetical protein	42.1 bits(21)	0, 49	21/21(100%)	0/21(0%)	Plus/Plus	
