# Supplementary figures and images for: Comparative genomics of Lentilactobacillus buchneri reveals strain-level hyperdiversity and broad-spectrum CRISPR immunity against human and livestock gut phages
Source: PLoS One. 2025 Jun 10;20(6):e0325832. doi: 10.1371/journal.pone.0325832 (PMC12151389; doi:10.1371/journal.pone.0325832)

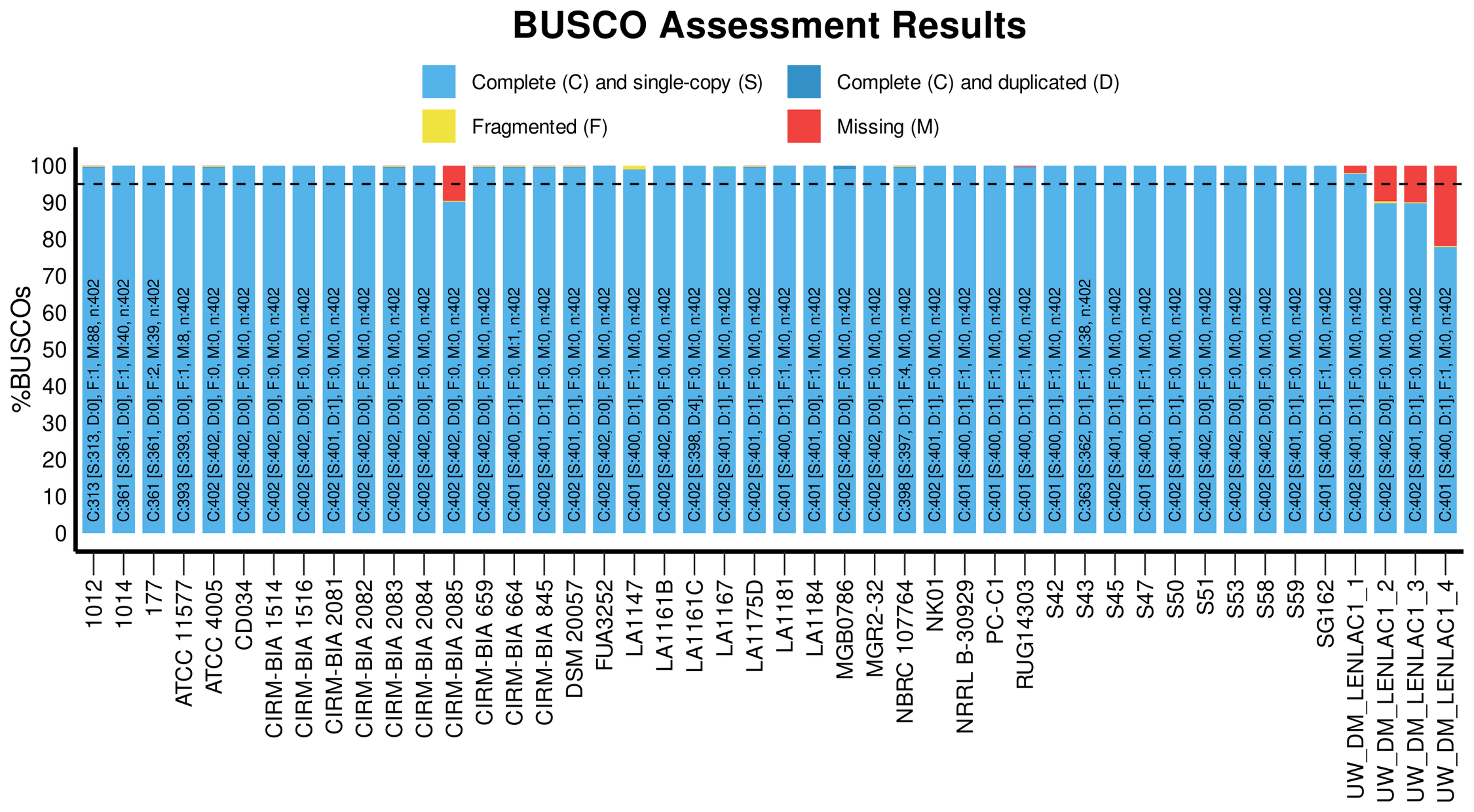

Supplement: S1 Fig — (TIF) [file pone.0325832.s005.tif]

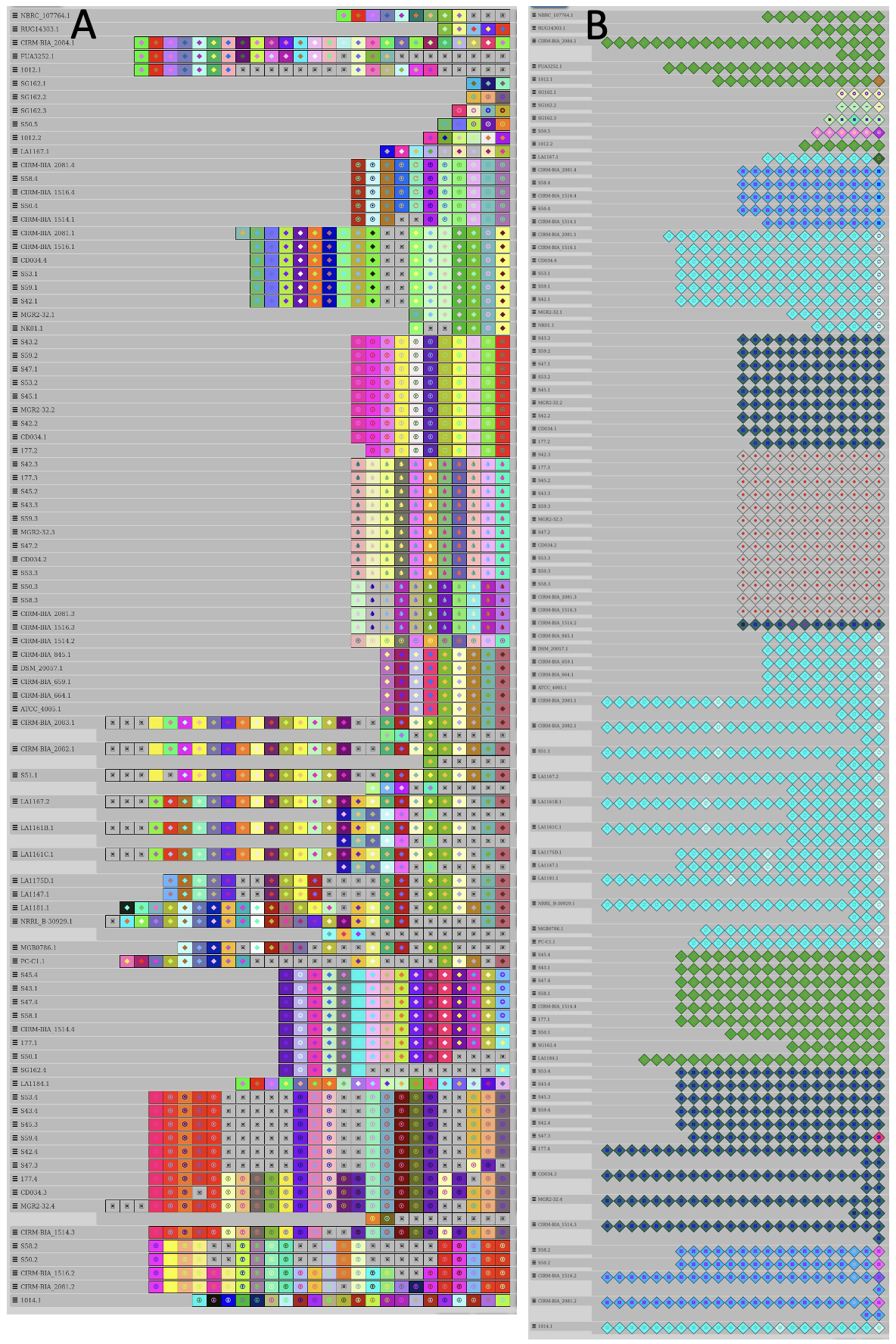

Supplement: S2 Fig — Each colored diamond represents a unique repeat, and each colored square represents a unique spacer in the CRISPR-Cas system. Grey “x” boxes showed a missing spacer. (TIF) [file pone.0325832.s006.tif]

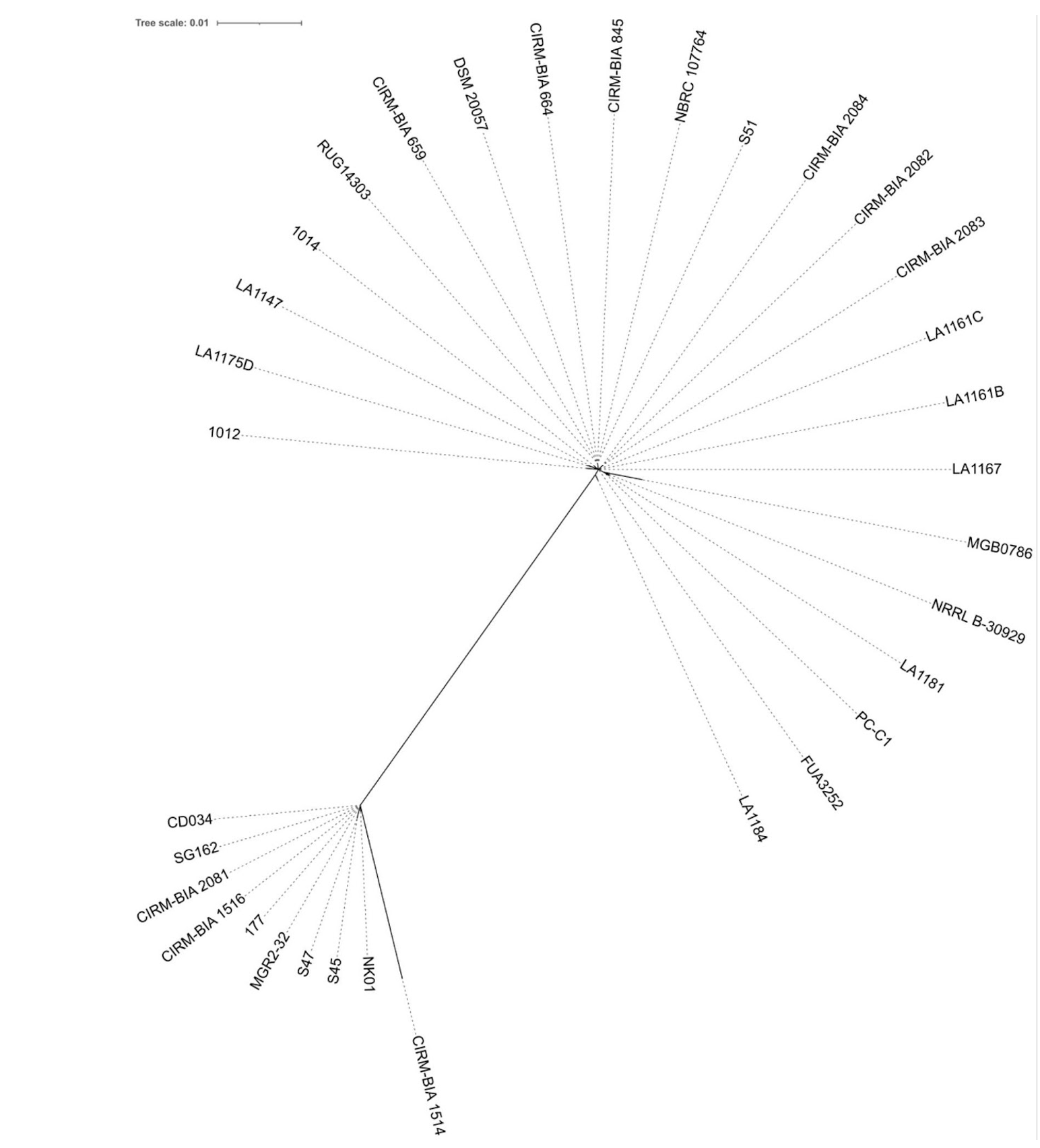

Supplement: S3 Fig — (TIF) [file pone.0325832.s007.tif]
