# Supplementary material for: Comparative genomics of Lentilactobacillus buchneri reveals strain-level hyperdiversity and broad-spectrum CRISPR immunity against human and livestock gut phages
Source: PLoS One. 2025 Jun 10;20(6):e0325832. doi: 10.1371/journal.pone.0325832 (PMC12151389; doi:10.1371/journal.pone.0325832)
Supplement: Attachment — Submitted filename: Clean_Copy_Revised_Lbuchneri_manuscript_300425_GoogleDocs.docx [file pone.0325832.s009.docx]

**Comparative Genomics of *Lentilactobacillus buchneri* Reveals Strain Level Hyperdiversity and Broad-spectrum CRISPR Immunity Against Human and Livestock Gut Phages**

Ismail Gumustop^1^, Ibrahim Genel^2^, Ibrahim Cagri Kurt^1^*, Fatih Ortakci^3^*

^1^Department of Molecular Biology and Genetics, Faculty of Arts and Sciences, Bogazici University, Istanbul, Turkiye

^2^School of Medicine, Koc University, Istanbul, Turkiye

^3^Department of Food Engineering, Faculty of Chemical and Metallurgical Engineering, Istanbul Technical University, Istanbul, Turkiye

***Corresponding Authors:** [**ibrahim.kurt@bogazici.edu.tr**](mailto:ibrahim.kurt@bogazici.edu.tr)**;** [**ortakci@itu.edu.tr**](mailto:ortakci@itu.edu.tr)

## Abstract

This study conducted a comparative genomic investigation of 40 strains of *Lentilactobacillus buchneri* isolated from various environments-including fermented foods, silage, cattle rumen, and the nasopharynx-to identify species-level diversity and assess their CRISPR immunity. The genomes were annotated and analyzed using tools such as Prokka and GET_HOMOLOGUES for phylogenetic reconstruction, as well as for the detection of CRISPR-Cas systems, prophages, and plasmids. An average genome size of 2.55 ±0.07 Mb, a GC content of 44.18±0.15%, and 2444±83 coding sequences were achieved. Prophages were found in all strains except for two, while 17 strains contained plasmids. No genes associated with bacteriocins were identified. CRISPR analysis revealed the presence of 42 Type II-A and 45 Type I-E systems, with each strain having at least one Type II-A system (~ 2 systems per strain). Among the 33 tested strains, 29 encoded complete LbCas9 proteins, consisting of 1371 amino acids. In-silico analysis of PAM in Type II-A systems revealed a 5’-DNAWDHV-3’ motif, with a noted preference for 5’-AAAA-3’ at positions 3-6. The spacers found in CRISPR arrays targeted proteins involved in plasmid mobilization as well as components of phage tails, indicating their roles in inhibiting horizontal gene transfer and providing defense against phages. Remarkably, 27 spacers from 24 strains were found to match phages associated with human gut microbiomes, with several showing the ability to cross-target phages from livestock, kefir, and wastewater. This research expands the genomic understanding of *L. buchneri* from 10 to 40 genomes, uncovering the dynamics of CRISPR-phage co-evolution. The specificity of the identified CRISPR systems and the broad-spectrum activity of the spacers highlight their potential for biotechnological uses, including CRISPR-based genome editing (referred to as CRISPRization) and phage therapy in fermentation processes. These findings improve our understanding of the ecological adaptability of *L. buchneri* and lay a groundwork for utilizing its CRISPR immunity in both industrial and therapeutic contexts.

**Key Words:** Comparative genomics, strain diversity, CRISPR, phage, immunity

## Introduction

*Lentilactobacillus buchneri* (*L. buchneri*) named after German microbiologist E. Buchner, is an obligatory heterofermentative lactic acid bacteria species that was previously known as *Bacillus buchneri*. *L. buchneri* was repeatedly isolated from several types of hard cheese and spreadable cheese namely Canestrato Pugliese and Ricotta Forte, respectively. The reference strain for this species is *L. buchneri* ATCC 4005, which was isolated from tomato pulp [[1,2]](https://www.zotero.org/google-docs/?NIgOw0).

High similarities were identified across *Levilactobacillus brevis* and *L. buchneri* such as similar isolation sources achieved for both species. *L. buchneri* manifests as chained short rods or single morphologies with rounded ends with a size of 0.7-1.0 × 2-4 μm under the light microscope. It grows in a wide range of temperatures (i.e., 15 to 37°C) but no growth occurs at 45°C. It has a GC content (mol%) of between 44-46% and contains lysine-d-aspartyl-type in its peptidoglycan layer. *L. buchneri* strains’ xylose, sucrose, lactose, galactose, raffinose, and esculin fermentation abilities vary from strain to strain. Two major unique phenotypic characteristics of *L. buchneri* that distinguish this species from *Levilactobacillus brevis* are the melezitose fermentation capability and the comparably slow migration of dual lactate dehydrogenase enzymes [[1]](https://www.zotero.org/google-docs/?wItkl5).

Fermented dairy foods are potential reservoirs for biogenic amines because of their rich amino acid content and possible metabolism of amino acids by adventitious non-starter lactic acid bacteria (LAB) during cheese making and ripening. Although primary microbes associated with biogenic amine formation are Enterobacteriaceae and *Pseudomonas*, the main culprits for biogenic amine production are LAB that belongs to lactobacilli, *Lactococcus*, *Leuconostoc*, *Streptococcus*, and *Enterococcus*. Among lactobacilli, the main biogenic amine formers are *L. buchneri*, *L. parabuchneri*, *Latilactobacillus curvatus*, *Lactobacillus helveticus*, *Limosilactobacillus vaginalis*, and *Levilactobacillus brevis*. It was reported that *L. buchneri* and *L. parabuchneri* can form histamine at refrigerator temperatures [[3,4]](https://www.zotero.org/google-docs/?JAgEtF).

*Lentilactobacillus buchneri* is an approved and heavily used starter culture to produce silage material for animal feed. One of the main reasons for *L. buchneri*’s popularity as a silage inoculant is because of its ability to increase the aerobic stability of silage material by diminishing the proliferation of fungi. Although homofermentative silage inoculants for example *Lactobacillus acidophilus*, *Lactiplantibacillus plantarum*, *Pediococcus cerevisiae*, *Pediococcus acidilactici*, and *Enterococcus faecium* are also being used to produce lactic acid, resulting silage fermentation product could be less stable under aerobic storage conditions as opposed to those products that have not been inoculated. It was reported that this is perhaps due to the metabolization of lactate by fungi species when the product is exposed to oxygen. Because *L. buchneri* is a heterofermentative LAB species, it produces acetate in addition to lactate which yields higher aerobic stability perhaps due to the stronger inhibitory effect of acetate against yeast and molds [[5]](https://www.zotero.org/google-docs/?Wnhufu).

Despite *L. buchneri* occupying a variety of different ecologies and has been used as a silage inoculant, there are limited studies on this species with regards to comparative genomic characterization for uncovering its strain level differences. To better understand species diversity at the strain level and CRISPR immunome of *L. buhneri* against human and livestock phages, we performed an extensive comparative genomic analysis of all strains that were deposited to NCBI GenBank. In the present work, we explored 40 strains including the reference strain ATCC 4005 through *in silico* genomic screening and analysis, significantly expanding upon the previous comparison of 10 strains [[6]](https://www.zotero.org/google-docs/?0slPJE).

## Methods

### Genome annotation

Whole-genome sequences of 46 *L. buchneri* strains were downloaded from NCBI with the following accession numbers of GCA_025186255.1, GCA_025186245.1, GCA_025186205.1, GCA_018314255.1, GCA_000298115.2, GCA_025194225.1, GCA_025194165.1, GCA_025194175.1, GCA_025194235.1, GCA_025194265.1, GCA_025194415.1, GCA_025194205.1, GCA_025212255.1, GCA_025190085.1, GCA_001434735.1, GCA_009495635.1, GCA_009495565.1, GCA_009495555.1, GCA_009495585.1, GCA_009495575.1, GCA_009495485.1, GCA_009495465.1, GCA_008369805.1, GCA_007992235.1, GCA_013167855.1, GCA_000211375.1, GCA_023507525.1, GCA_902797765.1, GCA_005048025.1, GCA_005049145.1, GCA_005047285.1, GCA_005048055.1, GCA_005047235.1, GCA_005049245.1, GCA_005047265.1, GCA_005047575.1, GCA_005049205.1, GCA_013167835.1, GCA_022651845.1.

Genome quality was assessed with BUSCO (version 5.4.3) [[7]](https://www.zotero.org/google-docs/?J0Ue9J) with lactobacillales_odb10 lineage dataset. Genomic annotation of sequences was accomplished using Prokka (version 1.14.5) with the following flag: --kingdom Bacteria. Roary was used to analyze GFF outputs from Prokka to investigate core and pangenome with the following flags: -e -n -v -r.

### Comparative genomics of *L. buchneri*

The evaluation of genomic similarity across forty *L. buchneri* genomes were conducted by using Jaccard distance using the prabclus package [[8]](https://www.zotero.org/google-docs/?HJyBci) according to the absence or presence of genes. The resulting data were processed through Principal Coordinate Analysis (PCoA) using R (version 4.1.1) [[9,10]](https://www.zotero.org/google-docs/?gBiLXf) to explore the relatedness among genomes.

The output of GFF files was analyzed using Roary (version 3.13.0) [[11]](https://www.zotero.org/google-docs/?YO6E9D) with flags "-e -n -v -r," which allowed us to analyze the pan- and core-genomes of the bacteria and compare the presence or absence of specific genes, including *hdcA* and *hisS*. The lower end BLASTp identity was set to 95% in Roary to secure accuracy. To explore whether the *L. buchneri* pangenome is open, we used the micropan package [[12]](https://www.zotero.org/google-docs/?6uKari) with 10,000 permutations to fit Heap's law model. These computational methods allowed us to get perception against the genetic makeup of *L. buchneri* and the variations of its pangenome.

The Single Nucleotide Polymorphisms (SNP) exist in the core genome were identified using snippy tool [[13]](https://www.zotero.org/google-docs/?iYV1si). In addition, core genome SNPs based phylogenetic tree was constructed using Clustal Omega [[14,15]](https://www.zotero.org/google-docs/?Qdna5T) and iTOL [[16]](https://www.zotero.org/google-docs/?c1YZBQ). We calculated average nucleotide identity (CDS ANI) using the GET_HOMOLOGUES [[17]](https://www.zotero.org/google-docs/?gm9x7z). Genomic islands were identified using GIPSy [[18]](https://www.zotero.org/google-docs/?DsgPip) by feeding GenBank annotation files from Prokka. The genomes were aligned and visualized using the BLAST Ring Image Generator (BRIG) software with DSM 20001 as the reference genome, using the BLASTn algorithm with a lower identity threshold of 70% and a higher identity threshold of 90% [[18,19]](https://www.zotero.org/google-docs/?Ahpnnk).

We used dbCAN2 meta server to annotate CAZymes (Carbohydrate Active Enzymes) encompassed by *L. buchneri* genomes. The CAZy annotation database (v11) was obtained after which HMMER (version 3.1b2) was applied for annotation of CAZyme domains [[20,21]](https://www.zotero.org/google-docs/?c5Z5m8). The CAZyme annotation outputs were filtered per default coverage thresholds and e-values by using dbCAN2. Next, CAZyme families were utilized to categorize the *L. buchneri* isolates. To identify putative bacteriocin biosynthesis related genes, we used BAGEL4 [[22]](https://www.zotero.org/google-docs/?Mk7t7c). Plasmids were detected by using PLSDB [[23,24]](https://www.zotero.org/google-docs/?7MZPAM). Prophages and metabolic islands were identified using PHASTEST [[25]](https://www.zotero.org/google-docs/?EA6USM) and GIPSy [[18]](https://www.zotero.org/google-docs/?SybA4F), respectively.

### CRISPR

CRISPRviz [[26]](https://www.zotero.org/google-docs/?0TCu9Q) was used to identify and visualize CRISPR loci in 40 strains. Types of the loci are determined by CRISPRCasFinder [[27]](https://www.zotero.org/google-docs/?Igiabc) and CRISPRClassify [[28]](https://www.zotero.org/google-docs/?gKXrbc). Open reading frames within +/-20kb of each array are predicted with Prodigal v2.6.3 [[29]](https://www.zotero.org/google-docs/?FEBvlf). hmmsearch function of hmmer v3.3.2 [[21]](https://www.zotero.org/google-docs/?fZkdvM) (HMM alignment coverage >70%, bitscore >= 40, E-value < 1e-5) was used to annotate Cas proteins with curated HMM profiles accessed from [[30]](https://www.zotero.org/google-docs/?pRc7N4), proteins predicted to be partial by Prodigal [[29]](https://www.zotero.org/google-docs/?dbsWpv) were removed during this process.

To analyze the relationship between diverse Cas9s, proteins named Cas9 (marked as non-fragment and longer than 800 aa) are downloaded from UniProt [[31]](https://www.zotero.org/google-docs/?aJXpic), sequences of commonly used Cas9s, and 33 cas9 from 40 strains were used for multiple sequence alignment with MAFFT v7.526 [[32]](https://www.zotero.org/google-docs/?J7Amgn). The phylogenetic tree of this alignment was generated with FastTree v2.1.11 [[33]](https://www.zotero.org/google-docs/?CK7yXO). While only focusing on the phylogenetic relationship between 33 cas9s from 40 strains, the same tools [[32,33]](https://www.zotero.org/google-docs/?FgcslT) and CRISPRviz [[26]](https://www.zotero.org/google-docs/?yESIlM) spacer and repeat results were used. To identify targets of the spacers, blastn [[34]](https://www.zotero.org/google-docs/?xZrmTg) results of PAMPredict v1.0.2 [[35]](https://www.zotero.org/google-docs/?ckAb2K), retrieved from searches against IMG/VR v4 [[36]](https://www.zotero.org/google-docs/?sk7VHq) and IMG/PR [[37]](https://www.zotero.org/google-docs/?GjvNzw), were examined. Megablast [[34]](https://www.zotero.org/google-docs/?UdCMd1) search against the NCBI nt database [[38]](https://www.zotero.org/google-docs/?Me7oPl) was performed to find out the top CDS hits of spacers. To predict the respective PAMs of 2 protein clusters, unique spacers belonging to the same clusters were gathered and were provided as input to CRISPRUtils [[39]](https://www.zotero.org/google-docs/?G12v7E).

## Results

### Genomic Characteristics

Forty-six *L. buchneri* genomes were downloaded from NCBI GenBank. All genomes were subjected to BUSCO [[7]](https://www.zotero.org/google-docs/?7g80fP) analysis to determine the completeness of the genomes (**S1 Figure**). Five genomes (i.e., CIRM-BIA 2085, UW_DM_LENLAC1_1, UW_DM_LENLAC1_2, UW_DM_LENLAC1_3, and UW_DM_LENLAC1_4) were excluded from further analysis due to showing below threshold level (i.e., 98%) completeness. The ATCC 11577 genome was discarded due to incorrect taxonomic assignment of genome assembly. **Table 1** represents forty *L. buchneri* strains isolated from different ecologies such as silage, fermented dough, grape must, pickles, cheese, kimchi, ethanol production facility, cattle rumen, and clinical samples. Whole-genome sequence statistics of *L. buchneri* isolates showed that the average genome size was 2.55 ± 0.08 Mb, GC content was 44.18 ± 0.19%, and number of CDS was 2443 ± 84.27, which are in alignment with the reference strain ATCC 4005.

**Table 1.** Whole-Genome assembly statistics of each of 40 *L. buchneri* genomes

| **Strain** | **Isolation Source** | **Assembly** | **Sequencing Technology** | **Size (Mb)** | **GC%** | **CDS** |
| --- | --- | --- | --- | --- | --- | --- |
| 177 | Mais silage | GCA_025186255.1 | Illumina 2x125 | 2.52 | 44.2 | 2406 |
| 1012 | Injera fermented dough | GCA_025186245.1 | Illumina 2x125 | 2.56 | 44.1 | 2554 |
| 1014 | Injera fermented dough | GCA_025186205.1 | Illumina 2x125 | 2.47 | 44.3 | 2407 |
| ATCC 4005 | Tomato pulp | GCA_018314255.1 | PacBio RSII; Illumina HiSeq | 2.54 | 44.4 | 2414 |
| CD034 | Stable grass silage | GCA_000298115.2 | 454 | 2.56 | 44.2 | 2425 |
| CIRM-BIA 1514 | Silage | GCA_025194225.1 | Illumina 2x125 | 2.54 | 44.2 | 2433 |
| CIRM-BIA 1516 | Silage | GCA_025194165.1 | Illumina 2x125 | 2.58 | 44.1 | 2479 |
| CIRM-BIA 2081 | Grape must | GCA_025194175.1 | Illumina 2x125 | 2.63 | 44.0 | 2508 |
| CIRM-BIA 2082 | Artisanal fermented pickles | GCA_025194235.1 | Illumina 2x125 | 2.54 | 44.2 | 2440 |
| CIRM-BIA 2083 | Artisanal fermented pickles | GCA_025194265.1 | Illumina 2x125 | 2.52 | 44.3 | 2411 |
| CIRM-BIA 2084 | Artisanal fermented pickles | GCA_025194415.1 | Illumina 2x125 | 2.57 | 44.0 | 2473 |
| CIRM-BIA 659 | Tomato pulp | GCA_025194205.1 | Illumina 2x125 | 2.50 | 44.2 | 2402 |
| CIRM-BIA 664 | Conjunctiva | GCA_025212255.1 | Illumina 2x125 | 2.50 | 44.3 | 2403 |
| CIRM-BIA 845 | Cheese (domiati) | GCA_025190085.1 | Illumina 2x125 | 2.50 | 44.3 | 2399 |
| DSM 20057 | Tomato pulp | GCA_001434735.1 | Illumina MiSeq; Illumina HiSeq | 2.45 | 44.4 | 2356 |
| FUA3252 | A fermented sorghum product | GCA_031629455.1 | Oxford Nanopore MinION | 2.60 | 44.2 | 2483 |
| LA1147 | Reduced NaCl fermented cucumber spoilage | GCA_009495635.1 | Illumina HiSeq | 2.62 | 44.0 | 2508 |
| LA1161B | Anaerobic fermented cucumber slurry spoilage | GCA_009495565.1 | Illumina HiSeq | 2.62 | 43.9 | 2534 |
| LA1161C | Anaerobic fermented cucumber slurry spoilage | GCA_009495555.1 | Illumina HiSeq | 2.57 | 44.1 | 2470 |
| LA1167 | Anaerobic fermented cucumber slurry spoilage | GCA_009495585.1 | Illumina HiSeq | 2.62 | 44.0 | 2530 |
| LA1175D | Reduced NaCl fermented cucumber spoilage | GCA_009495575.1 | Illumina HiSeq | 2.68 | 44.0 | 2573 |
| LA1181 | Reduced NaCl fermented cucumber spoilage | GCA_009495485.1 | Illumina HiSeq | 2.63 | 44.0 | 2538 |
| LA1184 | Reduced NaCl fermented cucumber spoilage | GCA_009495465.1 | Illumina HiSeq and PacBio | 2.76 | 44.0 | 2690 |
| MGB0786 | Kimchi | GCA_008369805.1 | PacBio RSII | 2.58 | 44.3 | 2512 |
| MGR2-32 | Grass silage | GCA_033096565.1 | illumina NovaSeq; PromethION | 2.54 | 44.3 | 2393 |
| NBRC 107764 | Tomato pulp | GCA_007992235.1 | Illumina HiSeq 1000 | 2.49 | 44.2 | 2405 |
| NK01 | Silage | GCA_013167855.1 | Illumina MiSeq | 2.65 | 43.9 | 2513 |
| NRRL B-30929 | Fuel ethanol production facility | GCA_000211375.1 | Sanger/454/Illumina | 2.59 | 44.2 | 2504 |
| PC-C1 | Fermented vegetables | GCA_023507525.1 | Illumina | 2.53 | 44.2 | 2437 |
| RUG14303 | Cattle rumen | GCA_902797765.1 | NA | 2.33 | 44.6 | 2213 |
| S42 | Nasopharynx | GCA_005048025.1 | Illumina MiSeq | 2.50 | 44.3 | 2363 |
| S43 | Nasopharynx | GCA_005049145.1 | Illumina MiSeq | 2.50 | 44.3 | 2362 |
| S45 | Nasopharynx | GCA_005047285.1 | Illumina MiSeq | 2.49 | 44.3 | 2362 |
| S47 | Nasopharynx | GCA_005048055.1 | Illumina MiSeq | 2.45 | 44.4 | 2306 |
| S50 | Nasopharynx | GCA_005047235.1 | Illumina MiSeq | 2.54 | 44.1 | 2419 |
| S51 | Nasopharynx | GCA_005049245.1 | Illumina MiSeq | 2.54 | 44.2 | 2446 |
| S53 | Nasopharynx | GCA_005047265.1 | Illumina MiSeq | 2.50 | 44.3 | 2362 |
| S58 | Nasopharynx | GCA_005047575.1 | Illumina MiSeq | 2.50 | 44.3 | 2361 |
| S59 | Nasopharynx | GCA_005049205.1 | Illumina MiSeq | 2.54 | 44.1 | 2418 |
| SG162 | Silage | GCA_013167835.1 | Illumina MiSeq | 2.65 | 44.0 | 2533 |

### Comparative Genomics

Forty *L. buchneri* genomes were processed through comparative genomic analysis to determine strain level uniqueness and CRISPR immunome traits in this species. Forty genomes including the reference strain ATCC 4005 were picked to construct a phylogenomic tree by using nucleotide sequence alignment of core genome based single nucleotide polymorphism (SNP) values of each individual strain (**Fig. 1**). Two major clades were identified according to SNP based phylogenetic evaluation. The first major clade from top-down mainly consisted of silage isolates and bovine nasopharynx. The exception to the first major clade was grape must that laid on in between two distinct ecological conditions. The second major clade was originating from fermented cucumber slurry spoilage, fermented pickles, bovine nasopharynx, cattle rumen, cheese, fuel ethanol production facility, fermented vegetables, injera fermented dough, kimchi, and tomato pulp. Noteworthy, the reference strain ATCC 4005 that was isolated from tomato pulp clustered with another tomato pulp isolate of NBRC 107764. It was interesting to note that bovine nasopharynx isolate of S51 excelled as an outlier strain that clustered with the second clade members. Overall, except for a few outliers, a phylogenetic relationship has been achieved according to isolation sources of *L. buchneri* genomes. For example, while silage isolates were primarily laid on the first major clade and closely located to each other on the phylogenetic tree, injera fermented dough isolates, and artisanal fermented pickles located according to their isolation sources in the second major clade. Moreover, fermented cucumber, fermented cucumber slurry spoilage, and fermented sorghum product isolates were closely located in the second major clade.

ANI (average nucleotide identity) based phylogenetic tree was constructed for quantitative analysis of similarity across forty *L. buchneri* strains (**Fig. 2**). The ANI results showed that all the strains screened belonged to *L. buchneri* due to the fact that the ANI value achieved was above 96%. The ANI values calculated ranged from 97.41-99.99%. The minimum ANI reached was seen among LA1184 and CIRM-BIA 2081, which were isolated from reduced NaCl fermented cucumber spoilage and grape must, respectively. On the contrary, one of the maximum ANI calculated was reached across the reference strain ATCC 4005 and DSM 20057, both were isolated from tomato pulp. Notably, strains S42 through S59 were isolated in the same year (2014) and in the same location (Lethbridge, Canada). In addition, they demonstrate a very high level of intergenomic similarity (highest ANI values), with the exception of S51 (**Fig. 1, Fig. 2**). It is likely that they might be the isolates of one strain that went through genetic drift that are circulating in bovine nasopharynx in this particular farm microenvironment.

We also constructed a PCoA plot based on Jaccard distance of presence and absence of genes exist in pangenome that were annotated with Prokka which is shown in **Fig. 3**. A clear clustering of strains had occurred on negative and positive sides of PCo1. On the positive side of PCo1, 16 isolates were accumulated half of which were belong to bovine nasopharynx and a quarter of which were belong to silage. On the negative side of PCo1, of the 24 strains condensed two third of which belonged to fermented foods including, fermented cucumber, pickles, fermented dough, kimchi, and cheese. The rest of the strains belonged to tomato pulp, conjunctiva, cattle rumen, nasopharynx, and fuel ethanol production facility.

BLAST Ring Image Generator (BRIG) was used to run comparative whole genome analysis of forty *L. buchneri* genomes against the reference strain ATCC 4005 (**Fig. 4**). Overall, comparison of putative coding sequences of all genomes versus reference strain ATCC 4005 revealed that a high identity percentage was seen with 70 to 100% identity as illustrated in **Fig. 4**. Declining GC percentage and decreasing BLAST identity showed three major gaps in the BRIG image. The first gap in the coverage is between the 280-310 kb region which contains a putative prophage. Similarly, the second and third gaps have a putative prophage region for each and located at 1160-1200 kp and 2470-2510 kb regions, respectively.

### Core- and Pangenome Analysis

Analysis of genomic conservation across forty *L. buchneri* genomes resulted in 31% of the pangenome being conserved within the 95% BLASTP identity threshold which accounts for the core genome (**Fig. 5A**). A total of 1006 genes, which corresponds to 17.3% of pangenome, forms shell genes whereas representation of cloud genes was achieved at 51.8% of total coding sequences suggesting phenotypic differences across *L. buchneri* strains analyzed [[40]](https://www.zotero.org/google-docs/?Bd72lk). To get a deeper understanding, random subsampling was implemented to construct each individual strains' trendlines of core- and pangenome (**Fig. 5B**). The size of the core genome was near to plateau right around the 20^th^ genome, whereas a flatline was not achieved with regards to size of pangenome. Because new genes are still in the process of being uncovered and the genome continues to increase, *L. buchneri* species has an open pangenome.

Among the total genes identified in the pangenome, 1792 of them were common for all genomes which represent the core genome. Across all *L. buchneri* genomes analyzed, 1012 was predicted to harbor 224 unique genes (**Fig. 5C**). The second and third largest unique gene counts (i.e. 164 and 132) were achieved by MGB0786 and MGB0786, respectively. On the contrary, *L. buchneri* S42 and S58 do not possess any unique genes compared to the other genomes screened.

**Functional Annotations**

The core and pangenomes were annotated by utilizing eggNOG-Mapper [[41,42]](https://www.zotero.org/google-docs/?EX1u6H). Functional COG categories of orthogroups were appointed according to the Database of Clusters of Orthologous Genes [[43]](https://www.zotero.org/google-docs/?9pDrgc). The largest core- and pangenome categories were associated with genes with function unknown (**Fig. 6A**). The second largest pangenome category was related to replication, recombination, and repair-associated genes. Amino acid transport and metabolism along with translation, ribosomal structure and biogenesis-related functional COG category forms the second and third abundance associated genes. Transcription as well as amino acid transport and metabolism had similar pan- and core genome related genes as they pertained to functional COG categories. Cell motility, secondary metabolites biosynthesis, transport and catabolism had the least number of core- and pangenome associated genes.

Analysis of CAZymes indicated three major distinct clades according to the number of genes associated with each family of CAZymes (**Fig. 6B**). The first clade from the very left was composed of six strains (i.e., CIRM-BIA 664, CIRM-BIA 659, CIRM-BIA 845, CIRM-BIA 2081, SG162, and NK01) that were isolated from conjunctiva, tomato pulp, cheese, grape must, rice grain silage, and silage, respectively. The second clade consisted of fifteen strains that were mostly isolated from either conjunctiva, fermented vegetables, tomato pulp, fermented pickles, fuel ethanol production facility, fermented cucumber spoilage, or silage. The third major clade contained the 48% of the strains including the reference strain ATCC 4005. The largest number of GH family enzymes encoding genes were discovered in SG162 isolated from rice grain silage. The second largest GH encoding genes were detected in CIRM-BIA 2081, CIRM-BIA 659, CIRM-BIA 664, and CIRM-BIA 845. The third largest number of GH related genes were found in NK01 and MGR2-32. The highest number of GT encoding genes were also found in NK01. CIRM-BIA 2084 ranked second according to the number of GT family enzyme encoding genes. The CE, AA, and CBM content of individual *L. buchneri* was comparable to each other and remarkably smaller compared to that of GH and GT family CAZymes.

A thorough screening of 40 *L. buchneri* genomes for the existence of histidine gene clusters was performed based on the presence or absence of the genes of *hdcA* (histidine decarboxylase), *hdcB* (histidine decarboxylase maturation protein), *hisS* (histidine tRNA ligase) and *hdcC* (histidine/histamine antiporter) revealed from Prokka. All strains were predicted to carry histidine tRNA ligase. LA1181 that was isolated from reduced NaCl fermented cucumber spoilage was the only strain among 40 *L. buchneri* strains analyzed that was predicted to harbor putative histidine decarboxylase gene. Still, remaining genes in *hdc* cluster (*hdcB* and *hdcC*) were not found in any of the strains tested.

### Mobile Genetic Elements

Forty *L. buchneri* isolates were explored for the existence of mobile genetic elements of prophages, plasmids, and CRISPR-Cas system. Genome evaluations for the existence of prophages and plasmids detected 72 intact prophages (**S2 Table**) and 32 plasmids (**S3 Table**). Of the 40 *L. buchneri* genomes analyzed, 38 of those harbored at least one intact prophage. On the other hand, 17 out of 40 *L. buchneri* strains were predicted to carry at least one plasmid. 76% of prophage containing genomes had carried more than one intact prophage. 41% of plasmid encoding *L. buchneri* genomes harbored multiple putative plasmids. 1014, LA1184, MGB0786, S51, and SG162 were predicted to carry the highest number of intact prophages (i.e. 3). No prophages were identified in LA1175D and RUG14303. Across 21 unique plasmids detected, NZ_CP073067.1 plasmid was the most abundant which accounts for ~19% of all plasmids determined. The second most abundant plasmid was NZ_CP065817.1 which was identified four times. The following plasmids of NC_016035.1, LR962096.1, and NZ_CP043613.1 were identified twice. The rest of the plasmids were detected once only and they account for half of the plasmids extracted. The highest number of plasmids were found in those strains isolated from reduced NaCl fermented cucumber spoilage and tomato pulp (i.e. 6 and 4, respectively).

The largest number of prophages was found in 1014, LA1184, MGB0786, S51, and SG162 (i.e. 3). The least number of prophages were detected in CIRM-BIA 2082, CIRM-BIA 2083, CIRM-BIA 2084, LA1147, S42, S43, S47, S53, and S58 (i.e. 1). The bovine nasopharynx isolate (i.e. S51) had the highest number of intact prophages in its genome (i.e., 14). This was followed by isolates of tomato pulp (i.e. 8) and anaerobic fermented cucumber slurry spoilage (i.e. 6). No bacteriocin biosynthesis related genes were found in any of 40 *L. buchneri* strains screened.

CRISPR-Cas systems enable bacteria and archaea to develop resistance against viruses, plasmids, and other foreign mobile genetic elements [[44]](https://www.zotero.org/google-docs/?bPS7Lq). Because *L. buchneri* strains have potential applications in the food industry, computational identification and comprehensive characterization of all CRISPR loci in the 40 strains were also performed. First, 40 genomes were screened using PILER-CR for the presence of potential CRISPR loci to identify direct repeats and spacers [[45]](https://www.zotero.org/google-docs/?In6HqX). Due to the fact that PILER-CR i) occasionally splits a single CRISPR array into multiple arrays, ii) outputs unusually short spacers (i.e. <10 nts) towards the ends of the arrays, iii) misses direct repeats with significant mismatches to the consensus direct repeat of the array, CRISPRviz tool was also employed in parallel [[26]](https://www.zotero.org/google-docs/?SGNOKG). CRISPRviz analysis for the direct repeats revealed 5 main clades with multiple strains and 5 unique clades with single members with occasional mutated repeats across the board in presumably historically older sequences at the ends of the arrays [[26,46]](https://www.zotero.org/google-docs/?x45c4Z) (**S2B Figure)**. In contrast, CRISPRviz analysis of spacers revealed 11 main clades with multiple strains clustered and 9 unique clades with single members (**S2A Figure**). CRISPR loci was then classified using two complementary approaches of CRISPRCasFinder and CRISPRClassify that are based on cas genes and direct repeats within loci, respectively [[27]](https://www.zotero.org/google-docs/?xCiozM). This was conducted to capture any CRISPR loci that might be lacking cas genes or arrays [[28]](https://www.zotero.org/google-docs/?Ajz1IV).

While there was a significant overlap between the tools, screening of cas genes around the identified arrays using protein homology identified several CRISPR systems lacking cas effector proteins. Eighty-eight CRISPR loci carried an array with or without cas proteins of which 84 were successfully assigned by CRISPRClassify (probability>0.90). Four loci with low prediction scores were then examined with CrisprCasFinder. CrisprCasFinder was able to make a prediction for 3 of 4 loci, as one of the locus was missing cas proteins that are prerequisite for CasFinder to function. In combination of both tools, 42 of Type II-A and 45 of Type I-E systems were characterized. Notably, all strains screened had carried at least one Type II-A system with a mean of ~2 total CRISPR systems per strain (**S1 Table**).

As the most abundant CRISPR system found in *L. buchneri* strains and the most widely studied platform among all CRISPR types, we further characterized the effector proteins of the Type II-A systems, namely *L. buchneri* Cas9s (LbCas9). Twenty-nine out of 33 LbCas9s from 33 strains were putatively complete and 1371 amino acids in length. However, LbCas9s from S45, S47, FUA3252, CIRM-BIA_1514 and MGB0786 strains were truncated from their C termini and 644, 644, 1007, 1152 and 1284 amino acids in length, respectively. When compared to 3366 different Cas9s cataloged in Uniprot, LbCas9s clustered separately from Cas9 effectors that have been widely characterized and employed, such as SpCas9, SaCas9, CjCas9, and NmeCas9s (**Fig. 7A**).

To elucidate whether the LbCas9 effectors of the members of the clades that formed based on direct repeat and spacer profiles previously were the same or similar, an unrooted phylogenetic analysis of the multiple protein sequence alignments of 33 LbCas9s from 33 strains were performed. Phylogenetic tree revealed two main clades carrying 10 or 23 members, respectively **(S3 Figure**). The genetic distance between the furthest two LbCas9s was 4.96%, reflecting a close and conserved CRISPR relationship between the strains in alignment with the ANI values (**Fig. 2**). While the direct repeat profiles did not completely segregate between the two LbCas9 clades, spacer profiles were clearly distinct (**Fig. 7B**).

Next, characterization of 40 *L. buchneri* strains’ CRISPR resistome was performed by focusing on the putative target protospacers of the spacers within our arrays with the goal of understanding the immune interactions among *L. buchneri*, bacteriophages, and other mobile genetic elements such as plasmids. PAMPredict tool was employed to search the spacers against 15,663,652 viral sequences from IMG/VR v4 and 699,973 plasmid sequences from IMG/PR [[36,37]](https://www.zotero.org/google-docs/?zf4722). The landscape revealed 30 clustered target profiles with diverse target organisms isolated from human and animal gut, sludge, kefir, anaerobic bioreactors, food waste, and wastewater microbial communities along with phages and plasmids from *Lactobacillus*, *Lentilactobacillus*, and *Agrilactobacilus* (**Fig. 8A**). Comprehensive protospacer analysis against the viral and plasmid databases also predicted the *in silico* PAM preference of the CRISPR systems of the larger LbCas9 clade. The statistical score of the smaller clade did not pass the threshold, as 5’-DNAWDHV-3’ motif downstream of protospacer with a relatively stronger preference of 5’-AAAA-3’ between the 3rd and 6th positions. This is in agreement with the previously reported PAM from a smaller cohort [[6]](https://www.zotero.org/google-docs/?KpGxWS) (**Fig. 8B**).

To further characterize the coding DNA sequences (CDS) that are putatively targeted by the spacers, we employed a megablast search against NCBI’s core nucleotide database [[38,47]](https://www.zotero.org/google-docs/?cErwuN). This analysis corroborated the observation that CRISPR systems within *L. buchneri* could provide resistance against plasmids and bacteriophages (**S4 Table**). The target plasmid CDS elements were concentrated on the mobilization proteins that are crucial for plasmid transfers between bacteria such as conjugal transfer protein (i.e., VirB4 of Type IV secretion system), mobA/mobL family protein, and mobilization protein 141 (mob141) [[48–50]](https://www.zotero.org/google-docs/?KPKHJu) (**S4 Table**). The target viral CDS elements were mostly condensed on phage tail components along with other phage genes such as phage tail tip lysozyme, tail length tape measure protein, tail completion protein, Baseplate J-like protein, portal protein, and Mg2+/Co2+ transport protein (**S4 Table**).

Analysis of CRISPR loci in *L. buchneri* strains identified 27 spacers that target four human gut phages (BK020950, BK021565, BK038207, BK046275) sourced from NCBI's database (**Fig. 8C, S4 Table**) [[51,52]](https://www.zotero.org/google-docs/?1Rmnbc). Validation through IMG databases revealed that spacer 3 of the second CRISPR locus in strain 1012 specifically targets phage BK038207 (from the human gut), which encodes a tail terminator protein (TrP) [[52]](https://www.zotero.org/google-docs/?RHYxac). The first spacer from the fourth CRISPR locus of strain S42, which targets BK021565, corresponded to phages found in bovine/sheep rumens and activated sludge, with the relevant area encoding a hypothetical protein that possesses an ABC-type ATPase domain and acetyltransferase, further indicated by Foldseek to include an AAA domain[[50–52]](https://www.zotero.org/google-docs/?SSB18O). These results emphasize the role of CRISPR in the targeting of phages across various ecological contexts.

The genetic similarity among phages derived from human, bovine, sheep, and sludge sources ranged from 50.92% to 51.34% for human-animal pairs and from 51.02% to 51.17% for human-sludge pairs, with sludge phages exhibiting a high degree of intra-group similarity at 99.60%, in contrast to animal gut phages which showed a similarity of 71.27% (**Fig. 8C**) [[50–52]](https://www.zotero.org/google-docs/?nwyeCs). Spacer 19 (strain S51) specifically targets phage BK020950 and a similar kefir phage with an 82.82% similarity, sharing conserved protospacer-PAM sequences that target a Sak4-like ssDNA annealing protein. Spacer 24 (LA1147) and spacer 6 (strain 1012) target different regions of the human gut phage BK046275: the portal protein and an unidentified hypothetical protein, respectively [[50-52]](https://www.zotero.org/google-docs/?FvqfsY). Even though the nucleotide similarity across phage sources is low (<52%), it is likely that CRISPR spacers recognize conserved functional domains (such as ATPase and portal proteins), highlighting the adaptability of CRISPR to divergent phages that possess essential genomic regions.

## Discussion

In this study, an in-depth comparative genomic exploration of forty *L. buchneri* strains isolated from tomato pulp, fermented cucumber, artisanal fermented pickles, fermented dough, silage, fermented sorghum product, cattle rumen, cheese, conjunctiva, fermented vegetables, fuel ethanol production facility, grape must, kimchi, and bovine nasopharynx was conducted. The genome sizes range between 2.33-2.76 Mb which are consistent with those of lactic acid bacteria species (i.e. 1.8 to 3.3 Mb). The average GC content achieved for *L. buchneri* was 44.18% which is in alignment with low GC LAB. The sequence analysis of whole genomes of *L. buchneri* detected single or multiple plasmids in 17 out of 40 genomes including the reference strain ATCC 4005. This further supports the hypothesis that LAB are well accustomed to their microniche by carrying plasmids in their genomes which could swiftly be acquired and transmitted during rapid environmental condition changes [[6]](https://www.zotero.org/google-docs/?Jt93wf).

We demonstrated the differences and similarities in phylogenetic trees based on core and pangenomes of *L. buchneri*. The discrepancies seen across core versus pangenome related phylogenetic trees perhaps could be attributed to the accessory genome including the presence of plasmids found in 42.5% of strains [[9]](https://www.zotero.org/google-docs/?AuNA82). The discrepancies could also be attributed to inaccurate assemblies [[53]](https://www.zotero.org/google-docs/?ESwy7k).

Pangenome outputs using Roary showed that *L. buchneri* has an open genome suggesting the functional diversity of this species. The open pangenome permits and facilitates the retrieval of genetic components from outside environments to cope with adverse environmental conditions and adapt [[54]](https://www.zotero.org/google-docs/?7M6iie). According to core orthogroups achieved in eggNOG-Mapper screening, a number of coding sequences were found to be associated with cell defense and repair which are crucial for growth and survival of microorganisms [[55]](https://www.zotero.org/google-docs/?5edeoY).

We detected a total of 72 intact prophages across all *L. buchneri* genomes with the exception of LA1175D and RUG14303, which belonged to fermented cucumber spoilage and cattle rumen microenvironments, respectively. Moreover, 22 genomic islands were identified according to fluctuating nucleotide sequence profiles. The existence of both mobile genetic elements of prophages and genomic islands imply the hallmark of the possible horizontal gene transfer events [[6]](https://www.zotero.org/google-docs/?KsvzAo). The percentage of unknown/hypothetical genes found in the whole genome was calculated to be at ~23%, which suggests that there is room for discovery for functional evaluation studies of *L. buchneri*. With about ~40% of coding sequences identified were conserved across 40 genomes screened, a significant amount of genetic diversity could be attributed to the accessory genome [[56]](https://www.zotero.org/google-docs/?emS49w). The existence of plasmids, genomic islands, and prophages proposed that mobile genetic elements are perhaps a significant genomic characteristic of *L. buchneri*.

Computational analysis of 40 *L. buchneri* genomes with regards CRISPR-Cas systems using CRISPRviz and CRISPRCasFinder showed that all the strains encoded a putative CRISPR system. The abundance of CRISPR in this species is higher than *Lactobacilli* and bacterial organisms overall, which suggests that *L. buchneri* is perhaps a remarkable reservoir for novel CRISPR based tools [[57]](https://www.zotero.org/google-docs/?q6pBI1). Type-IIA was the most abundant CRISPR-Cas system found in *L. buchneri* and this CRISPR type is the top candidate among all CRISPR systems for genetic engineering applications [[58]](https://www.zotero.org/google-docs/?Cp2FcK). Type-IE CRISPR-Cas system was also represented in ~43% of strains and this CRISPR toolbox can be reconfigurated for genetic modification applications especially in their native host [[59]](https://www.zotero.org/google-docs/?qwB2Dx).

Selective pressures within fermented environments (such as silage and kefir) influence the development of CRISPR-Cas systems in *L. buchneri*, evidenced by conserved patterns of spacers and direct repeats among different strains (**Fig. 7B**) [[60-63]](https://www.zotero.org/google-docs/?WKKCpU). These common structures likely indicate an adaptation to phages and mobile genetic elements, with the duplication of spacers occurring through horizontal gene transfer, repeated acquisitions, or selective benefits [[64]](https://www.zotero.org/google-docs/?QfgAmd). The phylogenetic grouping of LbCas9 alongside distinct CRISPR profiles (**Fig. 7B**) implies a coordinated evolutionary process, suggesting that these patterns could serve as potential phylogenetic indicators or tools for engineering strains resistant to phages [[65,66]](https://www.zotero.org/google-docs/?nP7nmv). Although the CRISPR adaptations in *L. buchneri* demonstrate ecological specialization within fermented environments, it remains essential to conduct functional validation for industrial use [[67,68]](https://www.zotero.org/google-docs/?sML5l5).

*L. buchneri*'s CRISPR spacers are effective against phages found in various environments, including human gut microbiome, kefir, rumen of livestock, and sewage (**Fig. 9**) [[69-72]](https://www.zotero.org/google-docs/?4t55Qs). Its utilization as a silage inoculant [[67,68]](https://www.zotero.org/google-docs/?yU6pgf) and its presence in crops such as tomatoes and cucumbers indicate ecological connections, likely facilitated by the use of manure fertilizers that transfer bacteria to soil and plants [[73,74]](https://www.zotero.org/google-docs/?6olKcz). Although existing databases tend to emphasize phages from the human gut, the independent targeting of similar phages by different strains (for example, from cucumber and fermented dough) suggests significant CRISPR-phage interactions [[75]](https://www.zotero.org/google-docs/?AYNruM). This highlights *L. buchneri*'s ability to adapt across various niches and the extensive defense capacity of its CRISPR system, which is relevant for food safety and microbial ecology.

Although the protospacer-spacer matches were statistically significant (e-value ≤ 0.001) for the 5 spacers that are 30 nts in length and putatively targeting the phages, there were up to three mismatches across 4 of the protospacers and a single PAM with a mismatch in the 5’-AAAA-3’ core. (**Fig. 8C, S4 Table**). For Cas9, the mismatches between spacer-protospacer that are neither in strongly conserved parts of PAM nor in the seed region (~10 nts upstream of PAM) are well tolerated [[76]](https://www.zotero.org/google-docs/?bVU2Pj). The mismatches could be due to: i) natural variations in phage strains due to genetic drift, ii) mutations in phage genomes that are positively selected under the CRISPR pressure, iii) the prophage stage of lifecycle that needs to be refractory to dsDNA breaks due to lethality in the host [[6]](https://www.zotero.org/google-docs/?loYlvU). It is likely possible that in the future, as more phages are sequenced and deposited to the databases, even stronger matches could be identified.

Because *L. buchneri* can also be detrimental to food bioprocessing due to causing food spoilage in particular at fermented cucumber industry, it is crucial to control the contamination of this organism to eliminate defects caused by this species [[77,78]](https://www.zotero.org/google-docs/?CM6H0V). In depth CRISPR spacer profiling could be used to eliminate the potential contamination of *L. buchneri* by designing phage therapy [[6]](https://www.zotero.org/google-docs/?k3dHk4). While phage therapy presents a promising strategy to control *L. buchneri*-induced spoilage in fermented foods, its ethical and environmental implications warrant careful consideration. Potential off-target effects on beneficial microbes, ecological disruptions, and the risk of horizontal gene transfer must be assessed [[79,80]](https://www.zotero.org/google-docs/?E9lo9T). The use of CRISPR spacer data to guide phage design may also raise concerns about accelerating microbial resistance dynamics [[81]](https://www.zotero.org/google-docs/?vHEHrn). Responsible application requires risk assessment, regulatory compliance, and transparency to ensure safe integration into food systems [[82]](https://www.zotero.org/google-docs/?y76auQ).

The extensive genome evaluation of *L. buchneri* revealed that this species carry genes as they pertain to CAZymes which are instrumental in carbohydrate synthesis and hydrolysis during fermentation. Although glycoside hydrolase, carbohydrate esterase, auxiliary activities, and carbohydrate binding modules were associated with degradation reactions, glycoside transferases participate in carbohydrate biosynthesis [[83]](https://www.zotero.org/google-docs/?FmdsGY). The abundance of GH related genes found in *L. buchneri* genomes suggest the carbohydrate fermentation capability of *L. buchneri* since sugar utilization is a significant demarcation of bacterium's functionality and creates a baseline for strain cultivation and selection [[84]](https://www.zotero.org/google-docs/?8yNQs1).

It has been reported that one of the most frequently encountered amine-producing bacteria present in sufficient quantities in dairy products is *L. buchneri*. For example, *L. buchneri* was found in Swiss cheese that had a significant concentration of histamine. Strains of *L. buchneri* isolated from Gouda cheese, were responsible for the production of histamine and tyramine, respectively [[85]](https://www.zotero.org/google-docs/?lDRRT5), implying potential mechanism of histidine to histamine conversion. Intriguingly, histidine decarboxylase, functional in catabolism of histamine from histidine, was only found in LA1181 that was isolated from reduced NaCl fermented cucumber spoilage. However, remaining two sets of genes (i.e. *hdcB* and *hdcC*) were missing, which might be due to evolutionary gene loss perhaps as a result of long proliferation of strain LA1181 under histidine-limited conditions of fermented cucumber [[70]](https://www.zotero.org/google-docs/?fqZoZz).

## Conclusion

The aim of the present study was to explore the strain level biodiversity within *L. buchneri* species by evaluating 40 complete genomes and further characterize the landscape of endogenous CRISPR-Cas loci for better utilization or control of this species in food bioprocessing conditions. We found that *L. buchneri* shows hyper variability across strains as revealed from phylogenetic trees. No clear relationship exists between isolation source and phylogenetics according to PcoA plot along with a similar carbohydrate-active enzyme profiles achieved regardless of origin’s of each strain imply a free-living lifestyle of this species. The relatively high frequency of intact prophages and plasmids found in the majority of strains perhaps demonstrate the plasticity of the genome. In contrast to presumed risk of histamine poisoning by *L. buchneri*, we did not come across the complete gene cassette required for histidine to histamine conversion across 40 genomes analyzed. This underlines the importance of functional testing of *L. bucheri* for histidine decarboxylase activity under *in vitro* conditions. A thorough examination of the CRISPR defense system reveals the co-evolutionary relationship between *L. buchneri* and the bacteriophages that share its natural environments and lifecycle. Understanding *L. buchneri*'s CRISPR-based immune responses opens up possibilities for biotechnological applications, including CRISPR manipulation and phage therapy.

### Acknowledgements

Not applicable.

### Authors’ Contributions

I.C.K and F.O. conceived and designed the study. I.G., I.G., I.C.K., and F.O. performed research. I.G., I.G., I.C.K., and F.O. collected the datasets and prepared the figures. I.G., I.G., I.C.K., and F.O. completed the analysis. I.C.K and F.O. wrote the manuscript. All authors edited and approved the final manuscript.

### Funding

This work has been supported by the Istanbul Technical University Scientific Research Projects Unit with grant number MGA-2023-45115. The funders had no role in study design, data collection and analysis, decision to publish, or preparation of the manuscript.

### Availability of data and materials

Genomes analyzed in the present study are available in NCBI with the following accession numbers: GCA_025186255.1, GCA_025186245.1, GCA_025186205.1, GCA_018314255.1, GCA_000298115.2, GCA_025194225.1, GCA_025194165.1, GCA_025194175.1, GCA_025194235.1, GCA_025194265.1, GCA_025194415.1, GCA_025194205.1, GCA_025212255.1, GCA_025190085.1, GCA_001434735.1, GCA_009495635.1, GCA_009495565.1, GCA_009495555.1, GCA_009495585.1, GCA_009495575.1, GCA_009495485.1, GCA_009495465.1, GCA_008369805.1, GCA_007992235.1, GCA_013167855.1, GCA_000211375.1, GCA_023507525.1, GCA_902797765.1, GCA_005048025.1, GCA_005049145.1, GCA_005047285.1, GCA_005048055.1, GCA_005047235.1, GCA_005049245.1, GCA_005047265.1, GCA_005047575.1, GCA_005049205.1, GCA_013167835.1, GCA_022651845.1.

## Declarations

### Ethics approval and consent to participate

Not applicable.

### Consent for publication

Not applicable.

### Competing interests

The authors declare no competing interests.

## References

[1.	Ibrahim SA. Lactic Acid Bacteria: Lactobacillus spp.: Other Species. Reference Module in Food Science. Elsevier; 2016. doi:10.1016/B978-0-08-100596-5.00857-X](https://www.zotero.org/google-docs/?34eSUY)

[2.	Sayers EW, Cavanaugh M, Clark K, Ostell J, Pruitt KD, Karsch-Mizrachi I. GenBank. Nucleic Acids Research. 2019;48: D84-D86. doi:10.1093/nar/gkz956](https://www.zotero.org/google-docs/?34eSUY)

[3.	Suzzi G, Perpetuini G, Tofalo R. Biogenic Amines. Third Edition. In: McSweeney PLH, McNamara JP, editors. Encyclopedia of Dairy Sciences (Third Edition). Third Edition. Oxford: Academic Press; 2022. pp. 95-102. doi:10.1016/B978-0-12-818766-1.00243-9](https://www.zotero.org/google-docs/?34eSUY)

[4.	Linares DM, del Río B, Ladero V, Martínez N, Fernández M, Martín MC, et al. Factors Influencing Biogenic Amines Accumulation in Dairy Products. Frontiers in Microbiology. 2012;Volume 3-2012. doi:10.3389/fmicb.2012.00180](https://www.zotero.org/google-docs/?34eSUY)

[5.	Combs DK, Hoffman PC. Lactobacillus buchneri for Silage Aerobic Stability. Focus on Forage. 2011;3. Available: https://fyi.extension.wisc.edu/forage/lactobacillus-buchneri-for-silage-aerobic-stability/](https://www.zotero.org/google-docs/?34eSUY)

[6.	Nethery MA, Henriksen ED, Daughtry KV, Johanningsmeier SD, Barrangou R. Comparative genomics of eight Lactobacillus buchneri strains isolated from food spoilage. BMC Genomics. 2019;20: 902. doi:10.1186/s12864-019-6274-0](https://www.zotero.org/google-docs/?34eSUY)

[7.	Manni M, Berkeley MR, Seppey M, Simão FA, Zdobnov EM. BUSCO Update: Novel and Streamlined Workflows along with Broader and Deeper Phylogenetic Coverage for Scoring of Eukaryotic, Prokaryotic, and Viral Genomes. Molecular Biology and Evolution. 2021;38: 4647–4654. doi:10.1093/molbev/msab199](https://www.zotero.org/google-docs/?34eSUY)

[8.	Hennig C, Hausdorf B. prabclus: Functions for Clustering and Testing of Presence-Absence, Abundance and Multilocus Genetic Data. 2024. Available: https://CRAN.R-project.org/package=prabclus](https://www.zotero.org/google-docs/?34eSUY)

[9.	Candeliere F, Raimondi S, Spampinato G, Tay MYF, Amaretti A, Schlundt J, et al. Comparative Genomics of Leuconostoc carnosum. Frontiers in Microbiology. 2021;Volume 11-2020. doi:10.3389/fmicb.2020.605127](https://www.zotero.org/google-docs/?34eSUY)

[10.	R Core Team. R: A Language and Environment for Statistical Computing. Vienna, Austria: R Foundation for Statistical Computing; 2024. Available: https://www.R-project.org/](https://www.zotero.org/google-docs/?34eSUY)

[11.	Page AJ, Cummins CA, Hunt M, Wong VK, Reuter S, Holden MTG, et al. Roary: rapid large-scale prokaryote pan genome analysis. Bioinformatics. 2015;31: 3691-3693. doi:10.1093/bioinformatics/btv421](https://www.zotero.org/google-docs/?34eSUY)

[12.	Snipen L, Liland KH. micropan: Microbial Pan-Genome Analysis. 2020. Available: https://CRAN.R-project.org/package=micropan](https://www.zotero.org/google-docs/?34eSUY)

[13.	Seemann T. Snippy: Rapid haploid variant calling and core genome alignment. Available: https://github.com/tseemann/snippy](https://www.zotero.org/google-docs/?34eSUY)

[14.	Sievers F, Wilm A, Dineen D, Gibson TJ, Karplus K, Li W, et al. Fast, scalable generation of high‐quality protein multiple sequence alignments using Clustal Omega. Molecular Systems Biology. 2011;7: 539. doi:10.1038/msb.2011.75](https://www.zotero.org/google-docs/?34eSUY)

[15.	Madeira F, Madhusoodanan N, Lee J, Eusebi A, Niewielska A, Tivey ARN, et al. The EMBL-EBI Job Dispatcher sequence analysis tools framework in 2024. Nucleic Acids Research. 2024;52: W521-W525. doi:10.1093/nar/gkae241](https://www.zotero.org/google-docs/?34eSUY)

[16.	Letunic I, Bork P. Interactive Tree Of Life (iTOL) v5: an online tool for phylogenetic tree display and annotation. Nucleic Acids Research. 2021;49: W293-W296. doi:10.1093/nar/gkab301](https://www.zotero.org/google-docs/?34eSUY)

[17.	Contreras-Moreira B, Vinuesa P. GET_HOMOLOGUES, a Versatile Software Package for Scalable and Robust Microbial Pangenome Analysis. Applied and Environmental Microbiology. 2013;79: 7696-7701. doi:10.1128/AEM.02411-13](https://www.zotero.org/google-docs/?34eSUY)

[18.	Soares SC, Geyik H, Ramos RTJ, Sá PHCG de, Barbosa EGV, Baumbach J, et al. GIPSy: Genomic island prediction software. Journal of Biotechnology. 2016;232: 2-11. doi:10.1016/j.jbiotec.2015.09.008](https://www.zotero.org/google-docs/?34eSUY)

[19.	Alikhan N-F, Petty NK, Ben Zakour NL, Beatson SA. BLAST Ring Image Generator (BRIG): simple prokaryote genome comparisons. BMC Genomics. 2011;12: 402. doi:10.1186/1471-2164-12-402](https://www.zotero.org/google-docs/?34eSUY)

[20.	Zhang H, Yohe T, Huang L, Entwistle S, Wu P, Yang Z, et al. dbCAN2: a meta server for automated carbohydrate-active enzyme annotation. Nucleic Acids Research. 2018;46: W95-W101. doi:10.1093/nar/gky418](https://www.zotero.org/google-docs/?34eSUY)

[21.	Eddy SR. Accelerated Profile HMM Searches. PLOS Computational Biology. 2011;7: 1-16. doi:10.1371/journal.pcbi.1002195](https://www.zotero.org/google-docs/?34eSUY)

[22.	van Heel AJ, de Jong A, Song C, Viel JH, Kok J, Kuipers OP. BAGEL4: a user-friendly web server to thoroughly mine RiPPs and bacteriocins. Nucleic Acids Research. 2018;46: W278–W281. doi:10.1093/nar/gky383](https://www.zotero.org/google-docs/?34eSUY)

[23.	Schmartz GP, Hartung A, Hirsch P, Kern F, Fehlmann T, Müller R, et al. PLSDB: advancing a comprehensive database of bacterial plasmids. Nucleic Acids Research. 2021;50: D273-D278. doi:10.1093/nar/gkab1111](https://www.zotero.org/google-docs/?34eSUY)

[24.	Galata V, Fehlmann T, Backes C, Keller A. PLSDB: a resource of complete bacterial plasmids. Nucleic Acids Research. 2018;47: D195–D202. doi:10.1093/nar/gky1050](https://www.zotero.org/google-docs/?34eSUY)

[25.	Wishart DS, Han S, Saha S, Oler E, Peters H, Grant JR, et al. PHASTEST: faster than PHASTER, better than PHAST. Nucleic Acids Research. 2023;51: W443–W450. doi:10.1093/nar/gkad382](https://www.zotero.org/google-docs/?34eSUY)

[26.	Nethery MA, and RB. CRISPR Visualizer: rapid identification and visualization of CRISPR loci via an automated high-throughput processing pipeline. RNA Biology. 2019;16: 577–584. doi:10.1080/15476286.2018.1493332](https://www.zotero.org/google-docs/?34eSUY)

[27.	Couvin D, Bernheim A, Toffano-Nioche C, Touchon M, Michalik J, Néron B, et al. CRISPRCasFinder, an update of CRISRFinder, includes a portable version, enhanced performance and integrates search for Cas proteins. Nucleic Acids Research. 2018;46: W246–W251. doi:10.1093/nar/gky425](https://www.zotero.org/google-docs/?34eSUY)

[28.	Nethery MA, Korvink M, Makarova KS, Wolf YI, Koonin EV, Barrangou R. CRISPRclassify: Repeat-Based Classification of CRISPR Loci. The CRISPR Journal. 2021;4: 558–574. doi:10.1089/crispr.2021.0021](https://www.zotero.org/google-docs/?34eSUY)

[29.	Hyatt D, Chen G-L, LoCascio PF, Land ML, Larimer FW, Hauser LJ. Prodigal: prokaryotic gene recognition and translation initiation site identification. BMC Bioinformatics. 2010;11: 119. doi:10.1186/1471-2105-11-119](https://www.zotero.org/google-docs/?34eSUY)

[30.	Makarova KS, Wolf YI, Alkhnbashi OS, Costa F, Shah SA, Saunders SJ, et al. An updated evolutionary classification of CRISPR–Cas systems. Nature Reviews Microbiology. 2015;13: 722–736. doi:10.1038/nrmicro3569](https://www.zotero.org/google-docs/?34eSUY)

[31.	Consortium TU. UniProt: the Universal Protein Knowledgebase in 2023. Nucleic Acids Research. 2022;51: D523–D531. doi:10.1093/nar/gkac1052](https://www.zotero.org/google-docs/?34eSUY)

[32.	Katoh K, Standley DM. MAFFT Multiple Sequence Alignment Software Version 7: Improvements in Performance and Usability. Molecular Biology and Evolution. 2013;30: 772–780. doi:10.1093/molbev/mst010](https://www.zotero.org/google-docs/?34eSUY)

[33.	Price MN, Dehal PS, Arkin AP. FastTree 2 – Approximately Maximum-Likelihood Trees for Large Alignments. PLOS ONE. 2010;5: 1–10. doi:10.1371/journal.pone.0009490](https://www.zotero.org/google-docs/?34eSUY)

[34.	Camacho C, Coulouris G, Avagyan V, Ma N, Papadopoulos J, Bealer K, et al. BLAST+: architecture and applications. BMC Bioinformatics. 2009;10: 421. doi:10.1186/1471-2105-10-421](https://www.zotero.org/google-docs/?34eSUY)

[35.	Ciciani M, Demozzi M, Pedrazzoli E, Visentin E, Pezzè L, Signorini LF, et al. Automated identification of sequence-tailored Cas9 proteins using massive metagenomic data. Nature Communications. 2022;13: 6474. doi:10.1038/s41467-022-34213-9](https://www.zotero.org/google-docs/?34eSUY)

[36.	Camargo AP, Nayfach S, Chen I-MA, Palaniappan K, Ratner A, Chu K, et al. IMG/VR v4: an expanded database of uncultivated virus genomes within a framework of extensive functional, taxonomic, and ecological metadata. Nucleic Acids Research. 2022;51: D733–D743. doi:10.1093/nar/gkac1037](https://www.zotero.org/google-docs/?34eSUY)

[37.	Camargo AP, Call L, Roux S, Nayfach S, Huntemann M, Palaniappan K, et al. IMG/PR: a database of plasmids from genomes and metagenomes with rich annotations and metadata. Nucleic Acids Research. 2023;52: D164–D173. doi:10.1093/nar/gkad964](https://www.zotero.org/google-docs/?34eSUY)

[38.	Sayers EW, Bolton EE, Brister JR, Canese K, Chan J, Comeau DC, et al. Database resources of the National Center for Biotechnology Information in 2023. Nucleic Acids Research. 2022;51: D29–D38. doi:10.1093/nar/gkac1032](https://www.zotero.org/google-docs/?34eSUY)

[39.	Nethery MA, Barrangou R. Chapter One - Predicting and visualizing features of CRISPR–Cas systems. In: Bailey S, editor. CRISPR-Cas Enzymes. Academic Press; 2019. pp. 1–25. doi:10.1016/bs.mie.2018.10.016](https://www.zotero.org/google-docs/?34eSUY)

[40.	Daughtry KV, Johanningsmeier SD, Sanozky-Dawes R, Klaenhammer TR, Barrangou R. Phenotypic and genotypic diversity of Lactobacillus buchneri strains isolated from spoiled, fermented cucumber. International Journal of Food Microbiology. 2018;280: 46–56. doi:10.1016/j.ijfoodmicro.2018.04.044](https://www.zotero.org/google-docs/?34eSUY)

[41.	Huerta-Cepas J, Szklarczyk D, Heller D, Hernández-Plaza A, Forslund SK, Cook H, et al. eggNOG 5.0: a hierarchical, functionally and phylogenetically annotated orthology resource based on 5090 organisms and 2502 viruses. Nucleic Acids Research. 2018;47: D309–D314. doi:10.1093/nar/gky1085](https://www.zotero.org/google-docs/?34eSUY)

[42.	Cantalapiedra CP, Hernández-Plaza A, Letunic I, Bork P, Huerta-Cepas J. eggNOG-mapper v2: Functional Annotation, Orthology Assignments, and Domain Prediction at the Metagenomic Scale. Molecular Biology and Evolution. 2021;38: 5825–5829. doi:10.1093/molbev/msab293](https://www.zotero.org/google-docs/?34eSUY)

[43.	Galperin MY, Wolf YI, Makarova KS, Vera Alvarez R, Landsman D, Koonin EV. COG database update: focus on microbial diversity, model organisms, and widespread pathogens. Nucleic Acids Research. 2020;49: D274–D281. doi:10.1093/nar/gkaa1018](https://www.zotero.org/google-docs/?34eSUY)

[44.	Barrangou R, Fremaux C, Deveau H, Richards M, Boyaval P, Moineau S, et al. CRISPR Provides Acquired Resistance Against Viruses in Prokaryotes. Science. 2007;315: 1709–1712. doi:10.1126/science.1138140](https://www.zotero.org/google-docs/?34eSUY)

[45.	Edgar RC. PILER-CR: Fast and accurate identification of CRISPR repeats. BMC Bioinformatics. 2007;8: 18. doi:10.1186/1471-2105-8-18](https://www.zotero.org/google-docs/?34eSUY)

[46.	Mojica FJM, Díez-Villaseñor C, García-Martínez J, Almendros C. Short motif sequences determine the targets of the prokaryotic CRISPR defence system. Microbiology. Microbiology Society; 2009. pp. 733–740. doi:https://doi.org/10.1099/mic.0.023960-0](https://www.zotero.org/google-docs/?34eSUY)

[47.	Morgulis A, Coulouris G, Raytselis Y, Madden TL, Agarwala R, Schäffer AA. Database indexing for production MegaBLAST searches. Bioinformatics. 2008;24: 1757–1764. doi:10.1093/bioinformatics/btn322](https://www.zotero.org/google-docs/?34eSUY)

[48.	Wallden K, Rivera-Calzada A, Waksman G. Microreview: Type IV secretion systems: versatility and diversity in function. Cellular Microbiology. 2010;12: 1203–1212. doi:10.1111/j.1462-5822.2010.01499.x](https://www.zotero.org/google-docs/?34eSUY)

[49.	Ramsay JP, Kwong SM, Murphy RJT, Eto KY, Price KJ, Nguyen QT, et al. An updated view of plasmid conjugation and mobilization in Staphylococcus. Mobile Genetic Elements. 2016;6: e1208317. doi:10.1080/2159256X.2016.1208317](https://www.zotero.org/google-docs/?34eSUY)

[50.	Francia MV, Varsaki A, Garcillán-Barcia MP, Latorre A, Drainas C, de la Cruz F. A classification scheme for mobilization regions of bacterial plasmids. FEMS Microbiology Reviews. 2004;28: 79–100. doi:10.1016/j.femsre.2003.09.001](https://www.zotero.org/google-docs/?34eSUY)

[51.	Nayfach S, Páez-Espino D, Call L, Low SJ, Sberro H, Ivanova NN, et al. Metagenomic compendium of 189,680 DNA viruses from the human gut microbiome. Nature Microbiology. 2021;6: 960–970. doi:10.1038/s41564-021-00928-6](https://www.zotero.org/google-docs/?34eSUY)

[52.	Tisza MJ, Buck CB. A catalog of tens of thousands of viruses from human metagenomes reveals hidden associations with chronic diseases. Proceedings of the National Academy of Sciences. 2021;118: e2023202118. doi:10.1073/pnas.2023202118](https://www.zotero.org/google-docs/?34eSUY)

[53.	Brandt K, Nethery MA, O’Flaherty S, Barrangou R. Genomic characterization of Lactobacillus fermentum DSM 20052. BMC Genomics. 2020;21: 328. doi:10.1186/s12864-020-6740-8](https://www.zotero.org/google-docs/?34eSUY)

[54.	Bazinet AL. Pan-genome and phylogeny of Bacillus cereus sensu lato. BMC Evolutionary Biology. 2017;17: 176. doi:10.1186/s12862-017-1020-1](https://www.zotero.org/google-docs/?34eSUY)

[55.	Oliveira FS, da Silva Rodrigues R, de Carvalho AF, Nero LA. Genomic Analyses of Pediococcus pentosaceus ST65ACC, a Bacteriocinogenic Strain Isolated from Artisanal Raw-Milk Cheese. Probiotics and Antimicrobial Proteins. 2023;15: 630–645. doi:10.1007/s12602-021-09894-1](https://www.zotero.org/google-docs/?34eSUY)

[56.	Gumustop I, Ortakci F. Comparative Genomics of Lentilactobacillus parabuchneri isolated from dairy, KEM complex, Makgeolli, and Saliva Microbiomes. BMC Genomics. 2022;23: 803. doi:10.1186/s12864-022-09053-y](https://www.zotero.org/google-docs/?34eSUY)

[57.	Sun Z, Harris HMB, McCann A, Guo C, Argimón S, Zhang W, et al. Expanding the biotechnology potential of lactobacilli through comparative genomics of 213 strains and associated genera. Nature Communications. 2015;6: 8322. doi:10.1038/ncomms9322](https://www.zotero.org/google-docs/?34eSUY)

[58.	Chylinski K, Makarova KS, Charpentier E, Koonin EV. Classification and evolution of type II CRISPR-Cas systems. Nucleic Acids Research. 2014;42: 6091–6105. doi:10.1093/nar/gku241](https://www.zotero.org/google-docs/?34eSUY)

[59.	Zheng Y, Li J, Wang B, Han J, Hao Y, Wang S, et al. Endogenous Type I CRISPR-Cas: From Foreign DNA Defense to Prokaryotic Engineering. Frontiers in Bioengineering and Biotechnology. 2020;Volume 8-2020. doi:10.3389/fbioe.2020.00062](https://www.zotero.org/google-docs/?34eSUY)

[60.	Abedon ST. Chapter 1 Phage Evolution and Ecology. Academic Press; 2009. pp. 1–45. doi:10.1016/S0065-2164(08)01001-0](https://www.zotero.org/google-docs/?34eSUY)

[61.	Barrangou R, Pijkeren J-P van. Exploiting CRISPR–Cas immune systems for genome editing in bacteria. Current Opinion in Biotechnology. 2016;37: 61–68. doi:10.1016/j.copbio.2015.10.003](https://www.zotero.org/google-docs/?34eSUY)

[62.	Millen AM, Horvath P, Boyaval P, Romero DA. Mobile CRISPR/Cas-Mediated Bacteriophage Resistance in Lactococcus lactis. PLOS ONE. 2012;7: 1–9. doi:10.1371/journal.pone.0051663](https://www.zotero.org/google-docs/?34eSUY)

[63.	Amundson KK, Roux S, Shelton JL, Wilkins MJ. Long-term CRISPR locus dynamics and stable host-virus co-existence in subsurface fractured shales. Current Biology. 2023;33: 3125-3135.e4. doi:10.1016/j.cub.2023.06.033](https://www.zotero.org/google-docs/?34eSUY)

[64.	Chakraborty S, Snijders AP, Chakravorty R, Ahmed M, Tarek AM, Hossain MA. Comparative network clustering of direct repeats (DRs) and cas genes confirms the possibility of the horizontal transfer of CRISPR locus among bacteria. Molecular Phylogenetics and Evolution. 2010;56: 878–887. doi:10.1016/j.ympev.2010.05.020](https://www.zotero.org/google-docs/?34eSUY)

[65.	Shariat N, Dudley EG. CRISPRs: Molecular Signatures Used for Pathogen Subtyping. Applied and Environmental Microbiology. 2014;80: 430–439. doi:10.1128/AEM.02790-13](https://www.zotero.org/google-docs/?34eSUY)

[66.	Doudna JA, Charpentier E. The new frontier of genome engineering with CRISPR-Cas9. Science. 2014;346: 1258096. doi:10.1126/science.1258096](https://www.zotero.org/google-docs/?34eSUY)

[67.	Elferink SJWHO, Krooneman J, Gottschal JC, Spoelstra SF, Faber F, Driehuis F. Anaerobic Conversion of Lactic Acid to Acetic Acid and 1,2-Propanediol by *Lactobacillus buchneri*. Applied and Environmental Microbiology. 2001;67: 125–132. doi:10.1128/AEM.67.1.125-132.2001](https://www.zotero.org/google-docs/?34eSUY)

[68.	Kleinschmit DH, Kung L. A Meta-Analysis of the Effects of Lactobacillus buchneri on the Fermentation and Aerobic Stability of Corn and Grass and Small-Grain Silages. Journal of Dairy Science. 2006;89: 4005–4013. doi:10.3168/jds.S0022-0302(06)72444-4](https://www.zotero.org/google-docs/?34eSUY)

[69.	Prado MR, Blandón LM, Vandenberghe LPS, Rodrigues C, Castro GR, Thomaz-Soccol V, et al. Milk kefir: composition, microbial cultures, biological activities, and related products. Frontiers in Microbiology. 2015;Volume 6-2015. doi:10.3389/fmicb.2015.01177](https://www.zotero.org/google-docs/?34eSUY)

[70.	Cheon M-J, Lim S-M, Lee N-K, Paik H-D. Probiotic Properties and Neuroprotective Effects of Lactobacillus buchneri KU200793 Isolated from Korean Fermented Foods. International Journal of Molecular Sciences. 2020;21. doi:10.3390/ijms21041227](https://www.zotero.org/google-docs/?34eSUY)

[71.	Amat S, Holman DB, Timsit E, Gzyl KE, Alexander TW. Draft Genome Sequences of 14 *Lactobacillus*, *Enterococcus*, and *Staphylococcus* Isolates from the Nasopharynx of Healthy Feedlot Cattle. Microbiology Resource Announcements. 2019;8: 10.1128/mra.00534-19. doi:10.1128/mra.00534-19](https://www.zotero.org/google-docs/?34eSUY)

[72.	Stewart RD, Auffret MD, Warr A, Walker AW, Roehe R, Watson M. Compendium of 4,941 rumen metagenome-assembled genomes for rumen microbiome biology and enzyme discovery. Nature Biotechnology. 2019;37: 953–961. doi:10.1038/s41587-019-0202-3](https://www.zotero.org/google-docs/?34eSUY)

[73.	Jaffar NS, Jawan R, Chong KP. The potential of lactic acid bacteria in mediating the control of plant diseases and plant growth stimulation in crop production - A mini review. Frontiers in Plant Science. 2023;Volume 13-2022. doi:10.3389/fpls.2022.1047945](https://www.zotero.org/google-docs/?34eSUY)

[74.	Gumustop I, Ortakci F. Analyzing the genetic diversity and biotechnological potential of Leuconostoc pseudomesenteroides by comparative genomics. Frontiers in Microbiology. 2023;Volume 13-2022. doi:10.3389/fmicb.2022.1074366](https://www.zotero.org/google-docs/?34eSUY)

[75.	Touchon M, Rocha EPC. The Small, Slow and Specialized CRISPR and Anti-CRISPR of Escherichia and Salmonella. PLOS ONE. 2010;5: 1–14. doi:10.1371/journal.pone.0011126](https://www.zotero.org/google-docs/?34eSUY)

[76.	Fu Y, Foden JA, Khayter C, Maeder ML, Reyon D, Joung JK, et al. High-frequency off-target mutagenesis induced by CRISPR-Cas nucleases in human cells. Nature Biotechnology. 2013;31: 822–826. doi:10.1038/nbt.2623](https://www.zotero.org/google-docs/?34eSUY)

[77.	Franco W, Pérez-Díaz IM, Johanningsmeier SD, McFeeters RF. Characteristics of Spoilage-Associated Secondary Cucumber Fermentation. Applied and Environmental Microbiology. 2012;78: 1273–1284. doi:10.1128/AEM.06605-11](https://www.zotero.org/google-docs/?34eSUY)

[78.	Sumner SS, Speckhard MW, Somers EB, Taylor SL. Isolation of histamine-producing Lactobacillus buchneri from Swiss cheese implicated in a food poisoning outbreak. Applied and Environmental Microbiology. 1985;50: 1094–1096. doi:10.1128/aem.50.4.1094-1096.1985](https://www.zotero.org/google-docs/?34eSUY)

[79.	Hagens S, Loessner MJ. Bacteriophage for Biocontrol of Foodborne Pathogens: Calculations and Considerations. Current Pharmaceutical Biotechnology. 2010;11: 58–68. doi:10.2174/138920110790725429](https://www.zotero.org/google-docs/?34eSUY)

[80.	Garvey M. Bacteriophages and Food Production: Biocontrol and Bio-Preservation Options for Food Safety. Antibiotics. 2022;11. doi:10.3390/antibiotics11101324](https://www.zotero.org/google-docs/?34eSUY)

[81.	Brüssow H. What is needed for phage therapy to become a reality in Western medicine? Virology. 2012;434: 138–142. doi:10.1016/j.virol.2012.09.015](https://www.zotero.org/google-docs/?34eSUY)

[82.	García P, Rodríguez L, Rodríguez A, Martínez B. Food biopreservation: promising strategies using bacteriocins, bacteriophages and endolysins. Trends in Food Science & Technology. 2010;21: 373–382. doi:10.1016/j.tifs.2010.04.010](https://www.zotero.org/google-docs/?34eSUY)

[83.	Lombard V, Golaconda Ramulu H, Drula E, Coutinho PM, Henrissat B. The carbohydrate-active enzymes database (CAZy) in 2013. Nucleic Acids Research. 2013;42: D490–D495. doi:10.1093/nar/gkt1178](https://www.zotero.org/google-docs/?34eSUY)

[84.	Jiang J, Yang B, Ross RP, Stanton C, Zhao J, Zhang H, et al. Comparative Genomics of Pediococcus pentosaceus Isolated From Different Niches Reveals Genetic Diversity in Carbohydrate Metabolism and Immune System. Frontiers in Microbiology. 2020;Volume 11-2020. doi:10.3389/fmicb.2020.00253](https://www.zotero.org/google-docs/?34eSUY)

[85.	Calasso M, Gobbetti M. Lactic Acid Bacteria | Lactobacillus spp.: Other Species. Second Edition. In: Fuquay JW, editor. Encyclopedia of Dairy Sciences (Second Edition). Second Edition. San Diego: Academic Press; 2011. pp. 125–131. doi:10.1016/B978-0-12-374407-4.00265-X](https://www.zotero.org/google-docs/?34eSUY)

**List of Figures**

**Fig. 1** SNP-based phylogenetic tree of 40 *L. buchneri* isolates and their isolation sources

**Fig. 2** Average nucleotide identity based heat map and clustering of 40 *L. buchneri* genomes. The color gradient from red to dark blue shows a higher trend in percent identity.

**Fig. 3** PCoA graph of Jaccard distances based on shared genes among 40 *L. buchneri* genomes analyzed. Each colored box shows a unique isolation source.

**Fig. 4** BLAST Ring Image Generator analysis of 39 *L. buchneri* genomes against reference genome ATCC 4005. The innermost ring shows the location of the genome. Prophages and genomic islands were depicted outside of the rings.

**Fig. 5** (A) Distribution of the coding sequences across core and pangenome. (B) Number of core genes (light green curve) vs pan genes (dark blue curve). (C) Flower plot of core and unique gene families of 40 *L. buchneri* isolates

**Fig. 6** (A) CAZyme heat map of 40 *L. buchneri* strains. The color gradient from lighter to darker colors represent the abundance of CAZymes found in each genome. GH: Glycoside hydrolase, GT: Glycosyltransferase, CE: Carbohydrate esterase, AA: Auxiliary activity, CBM: Carbohydrate binding module. R programming language [10] (version 4.1.1) was used to draw the heatmap. (B) Functional COG analysis across core and pangenomes of 40 *L. buchneri* strains. (Core genes (Red), Accessory genes (Dark blue).

**Fig. 7** (A) Phylogenetic tree of 3366 different Cas9s cataloged in Uniprot and 33 LbCas9s. Widely characterized effectors and LbCas9s are denoted with additional lines. (B) Phylogenetic tree of 33 LbCas9s based on primary amino acid sequence similarity. Colors denote different groups identified by CRISPRviz regarding repeat and spacer similarity.

**Fig. 8** (A) Heatmap of all spacers with significant hits, identified by PAMPredict, when searched against IMGVR and IMGPR databases. Target samples are binned into categories instead of displaying the individual strain information. Dendrograms show the relationships within spacers and hits. (B) *In silico* predicted PAM sequence as determined by the alignment of 7 different protospacer-flanking sequences. (C) The genomic context of 4 distinct phages from human gut microbiome targeted by spacers are shown. In sequence alignment, the top strands represent the phage (protospacer) and bottom strands represent the spacer sequences.

**Fig. 9** (A) Categorization of *L. buchneri* strains based on their sources of isolation. (B) Representative schematic of the potential lifecycle of *L. buchneri* strains starting as part of starter cultures used in kefir and silage. Bacteriophages that are targeted by CRISPR resistome are indicated by their sources of isolation. Vertical colored bars below phages represent spacer numbers and identities targeting each phage. (Phage, cucumber, tomato, stool, and sewage icons are sourced from flaticon.com)

**S1 Table.** CRISPRCasFinder results of L. buchneri genomes tested

**S2 Table.** Putative prophages predicted in L. buchneri strains using PHASTEST

**S3 Table.** Putative plasmids found in L. buchneri strains

**S4 Table.** Megablast search results showing best CDS hits targeted by spacers.

**S1 Figure.** BUSCO assessment results of 46 L. buchneri genome assemblies.

**S2 Figure.** Alignment of spacers (A) and repeats (B) of each detected CRISPR locus. Each colored diamond represents a unique repeat, and each colored square represents a unique spacer in the CRISPR-Cas system. Grey “x” boxes showed a missing spacer.

**S3 Figure.** Unrooted phylogenetic tree of 33 LbCas9s based on primary amino acid sequence similarity
